# Endocytic trafficking of GAS6–AXL complexes is associated with sustained AKT activation

**DOI:** 10.1007/s00018-022-04312-3

**Published:** 2022-05-27

**Authors:** Agata Poświata, Kamila Kozik, Marta Miączyńska, Daria Zdżalik-Bielecka

**Affiliations:** Laboratory of Cell Biology, International Institute of Molecular and Cell Biology, Warsaw, Poland

**Keywords:** AXL, GAS6, TAM receptors, Endocytosis, Recycling, SNX1

## Abstract

AXL, a TAM receptor tyrosine kinase (RTK), and its ligand growth arrest-specific 6 (GAS6) are implicated in cancer metastasis and drug resistance, and cellular entry of viruses. Given this, AXL is an attractive therapeutic target, and its inhibitors are being tested in cancer and COVID-19 clinical trials. Still, astonishingly little is known about intracellular mechanisms that control its function. Here, we characterized endocytosis of AXL, a process known to regulate intracellular functions of RTKs. Consistent with the notion that AXL is a primary receptor for GAS6, its depletion was sufficient to block GAS6 internalization. We discovered that upon receptor ligation, GAS6–AXL complexes were rapidly internalized via several endocytic pathways including both clathrin-mediated and clathrin-independent routes, among the latter the CLIC/GEEC pathway and macropinocytosis. The internalization of AXL was strictly dependent on its kinase activity. In comparison to other RTKs, AXL was endocytosed faster and the majority of the internalized receptor was not degraded but rather recycled via SNX1-positive endosomes. This trafficking pattern coincided with sustained AKT activation upon GAS6 stimulation. Specifically, reduced internalization of GAS6–AXL upon the CLIC/GEEC downregulation intensified, whereas impaired recycling due to depletion of SNX1 and SNX2 attenuated AKT signaling. Altogether, our data uncover the coupling between AXL endocytic trafficking and AKT signaling upon GAS6 stimulation. Moreover, our study provides a rationale for pharmacological inhibition of AXL in antiviral therapy as viruses utilize GAS6–AXL-triggered endocytosis to enter cells.

**Supplementary Information:**

The online version contains supplementary material available at 10.1007/s00018-022-04312-3.

## Introduction

AXL together with TYRO3 and MER represent TAM receptor tyrosine kinases (RTKs) that mediate phagocytic clearance of apoptotic cells and innate immune responses [1–4]. These receptors are activated by two vitamin K-dependent ligands, protein S (PROS1) which binds TYRO3 and MER, and growth arrested specific protein 6 (GAS6) which is postulated to activate all three TAMs [5–7]. TAMs have also been implicated in cancer, with AXL playing a prominent role in cancer progression [8–10]. AXL and/or GAS6 overexpression have been reported in multiple human cancers such as glioma [11], melanoma [12], breast [13], lung [14], and ovarian cancer [15], and high level of AXL was associated with increased tumor progression and poorer overall survival [16, 17]. AXL has also been linked to epithelial-to-mesenchymal transition (EMT)-like phenotype and acquired resistance to both conventional and targeted anti-cancer therapies [18, 19]. Given this, AXL is a promising therapeutic target, and R428 (bemcentinib), a first-in-class AXL kinase inhibitor, is currently being evaluated in phase 2 clinical trials for metastatic lung and triple-negative breast cancer, glioblastoma, and acute myeloid leukemia [20, 21].

Besides its role in carcinogenesis, AXL serves as an important receptor for the cellular entry of multiple viruses, including Lassa, Ebola, and ZIKA virus (ZIKV) [8, 22–25]. Importantly, TAM signaling appears to be involved in different stages of SARS-CoV-2 infection and progression of COVID-19 [26, 27]. A recent study of Wang et al*.* revealed that AXL serves as an entry receptor for SARS-CoV-2 in pulmonary and bronchial epithelial cells [28]. In fact, bemcentinib has been fast-tracked toward phase 2 clinical trials for the treatment of British COVID-19 patients under the ACCORD program (The Accelerating COVID-19 Research & Development Platform) [26, 29, 30].

As other RTKs, upon ligand binding, AXL becomes activated, autophosphorylated and triggers downstream signaling pathways such as those involving PI3K–AKT and ERK1/2 [31–33]. However, systematic analyses of AXL-activated intracellular processes and effectors have been reported only very recently. We revealed a proximity interactome of AXL in glioblastoma cells [31], while Abu-Thuraia et al. identified a phosphoproteome of AXL in breast cancer cells [34]. Both data sets showed a high degree of consistency and demonstrated an involvement of AXL in multiple processes involving actin remodeling. In line with this, GAS6-activated AXL induced the formation of peripheral and circular dorsal ruffles, macropinocytosis, and focal adhesion turnover, all of which depend on actin remodeling [31, 34]. Notably, the identified interactome and phosphoproteome of AXL were significantly enriched in endocytic proteins [31, 34].

Endocytosis is considered as an important organizer of cellular signaling induced by RTKs [35]. During endocytosis, RTKs activated by their cognate ligands are internalized into early endosomes from where they are sorted toward degradation in lysosomes or recycled back to the plasma membrane which terminates or sustains their signaling, respectively [36]. Moreover, RTKs can be internalized via distinct endocytic pathways, dependent or independent of clathrin that may affect signaling outcomes [35–40]. During clathrin-mediated endocytosis (CME), clathrin is recruited by adaptors, such as the AP-2 adaptor protein complex, EPS15/EPS15L1 or NUMB [41], and cargo is incorporated into clathrin-coated pits, which subsequently pinch off from the cell surface as clathrin-coated vesicles [42]. Their dissociation from the plasma membrane is catalyzed by dynamins (DNMs), large GTPases, with DNM2 playing a predominant role in most cell types [43, 44]. DNM2 is also involved in some pathways of clathrin-independent endocytosis (CIE) [45–47]. CIE is a general term for several uptake mechanisms that occur in the absence of clathrin; however, their classification is still under debate. Typically, they are distinguished based on the involvement of coat or regulatory proteins and generally depend on actin and actin-associated proteins [46–48]. Among CIE pathways, caveolin-dependent endocytosis operates through caveolae, flask-shaped structures formed by caveolins and cavins [49]. Another CIE pathway is mediated by flotillins which cluster on the plasma membrane forming microdomains that decorate membrane invaginations [50–52]. Whereas CME and caveolin-dependent endocytosis rely on dynamin for vesicle scission, both dynamin-dependent and -independent flotillin-mediated pathways were described [53]. Fast-endophilin-mediated endocytosis (FEME), another CIE route that operates on the leading edge of migrating cells, also requires dynamin that together with endophilins mediate scission of endophilin-positive assemblies [54, 55]. In contrast, macropinocytosis and the CDC42- and GRAF1-regulated clathrin-independent carriers (CLIC)/GPI-AP-enriched compartments (GEEC) pathway do not require dynamin [56].

Although endocytosis can modulate signaling activated by RTKs, the majority of our knowledge about this relationship is based on data obtained for one prototype RTK, namely epidermal growth factor receptor (EGFR) [37, 57], whereas endocytosis of AXL or the other TAMs has not been studied. Here, we dissect the routes of internalization and endocytic traffic of AXL, as the first TAM family member investigated in this respect.

## Results

### AXL and its kinase activity are necessary for internalization of GAS6–AXL complexes

To study endocytic trafficking of GAS6–AXL complexes, we established the following cell stimulation and staining procedure. We incubated serum-starved glioblastoma LN229 cells, which we previously used for the identification of AXL interactome [31], with purified Myc-tagged version of GAS6 (hereafter called GAS6) for various time periods. Then, fixed cells were stained with anti-Myc and anti-AXL antibodies to visualize the ligand and the receptor, respectively (e.g., Fig. S1A), and the resulting confocal images were quantitatively analyzed. Specifically, we measured the number and the integral fluorescence intensity of GAS6- and AXL-positive vesicles, as well as their colocalization with endocytic markers.

**Fig. 1:**
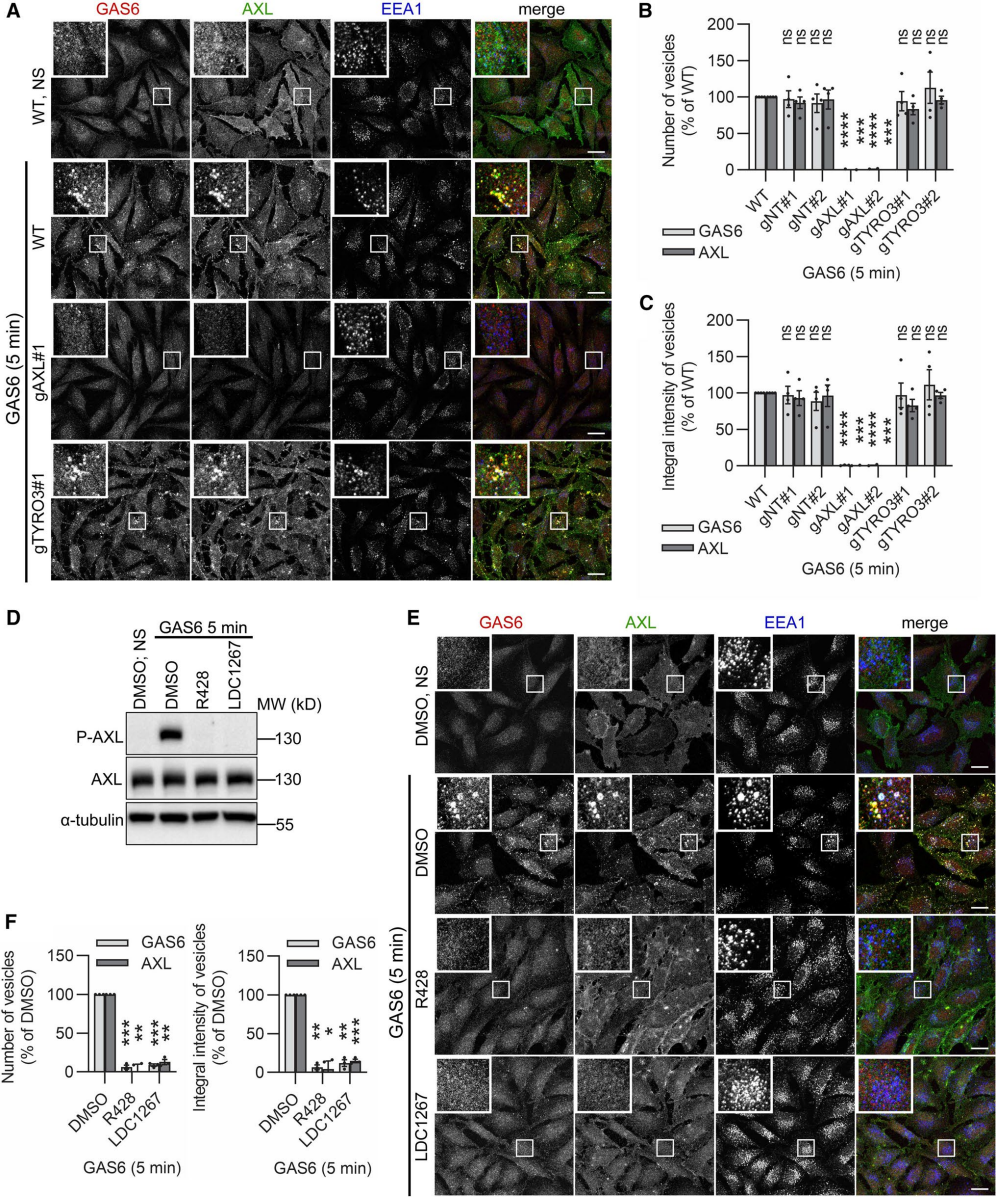
AXL and its kinase activity are required for GAS6-mediated internalization of GAS6–AXL complexes. **A** Confocal images showing GAS6–AXL internalization upon knockout of *AXL* and *TYRO3* in LN229 cells. Two gRNAs targeting *AXL* (gAXL#1 and gAXL#2) and *TYRO3* (gTYRO3#1 and TYRO3#2) were used. CRISPR-Cas9-edited LN229 cells with two non-targeting gRNAs (gNT#1 and gNT#2) served as controls. Serum-starved cells were stimulated with GAS6 for 5 min. **B**, **C** Quantification of number (B) and integral fluorescence intensity (**C**) of GAS6- and AXL-positive vesicles in CRISPR-Cas9-mediated knockouts of *AXL* and *TYRO3* (representative confocal images shown in (**A**), *n* = 4. Student’s one-sample *t* test, ***p ≤ 0.001, ****p ≤ 0.0001, *ns* non-significant (*p* > 0.05). **D** Western blot showing phosphorylation of AXL (P-AXL, Y702) after treatment of LN229 cells with AXL inhibitors. Serum-starved cells were pre-treated with R428 or LDC1267, or DMSO as a solvent control, and stimulated with GAS6 for 5 min. α-Tubulin served as a loading control. **E** Confocal images showing the internalization of GAS6–AXL complexes upon pharmacological inhibition of AXL. LN229 cells were treated as described in (**D**). **F** Quantification of number and integral fluorescence intensity of GAS6- and AXL-positive vesicles in LN229 cells after treatment with R428 and LDC1267 (representative confocal images shown in (**E**), *n* = 3. Student’s one-sample *t* test, *p ≤ 0,05, **p ≤ 0.01, ***p ≤ 0.001, *ns* non-significant (*p* > 0.05). Data information: Insets in confocal images show magnified views of boxed regions in the main images. Scale bars: 20 μm. For data quantification, approximately 150 cells were analyzed per experiment. Each dot represents data from one independent experiment, whereas bars represent the means ± SEM from *n* experiments. *WT* wild type LN229 cells, *NS* non-stimulated cells, *GAS6* GAS6-stimulated cells

Since LN229 cells express two TAMs, AXL and TYRO3 [31, 58], and GAS6 was proposed to function as a ligand for all three TAMs [5–7], we first assessed the involvement of AXL and TYRO3 in internalization of GAS6. To discriminate which of these receptors is required for GAS6 endocytosis, we measured its accumulation in the previously generated *AXL* and *TYRO3* knockout (KO) LN229 cells [31]. Immunostaining for early endosome antigen 1 (EEA1) was used to mark early endosomes. As shown in Fig. 1A–C and Fig. S1B-D, CRISPR-Cas9-mediated inactivation of *AXL*, but not of *TYRO3*, completely blocked the internalization of GAS6 in cells treated with the ligand for 5 or 10 min. These results were also confirmed by silencing of *AXL* and *TYRO3* by siRNA in LN229 cells (Fig. S2A–D). Importantly, siRNA-mediated inactivation of *AXL* was also sufficient to block the internalization of GAS6 in ovarian cancer SKOV3 cells that express all three TAM receptors (Fig. S2E–G) [59]. Next, we determined whether activation of the tyrosine kinase domain of AXL is required for GAS6-induced AXL internalization. To this end, we pre-treated LN229 cells with AXL inhibitors, R428 or LDC1267, prior to GAS6 stimulation. Both compounds inhibited AXL phosphorylation and internalization of GAS6 and AXL (Fig. 1D–F).

Taken together, our data show that AXL is the primary receptor for GAS6, and internalization of GAS6–AXL complexes depends on the kinase activity of AXL.

### GAS6 and AXL accumulate rapidly on endosomal structures

To characterize the course of GAS6–AXL endocytosis and to follow one single round of ligand–receptor internalization, we employed pulse-chase stimulation. To this end, we incubated serum-starved LN229 cells with GAS6 on ice, and after removing of unbound ligand, endocytosis was allowed to proceed at 37 °C for increasing time periods. As shown in Fig. S3A, GAS6 and AXL rapidly accumulated on vesicular structures. The number and integral intensity of fluorescence of GAS6- and AXL-positive vesicles peaked at 5 min of stimulation and thereafter quickly decreased reaching the level of unstimulated cells after 30 min of endocytosis (Fig. S3A-C). Importantly, AXL vesicles showed up to 50% colocalization with GAS6-containing vesicles, indicating that a substantial fraction of internalized AXL traffics through the endosomal system in a ligand-bound state (Fig. S3A and D). Similar kinetics of endocytosis of GAS6–AXL complexes was observed in SKOV3 cells (Fig. S4A–D).

**Fig. 2:**
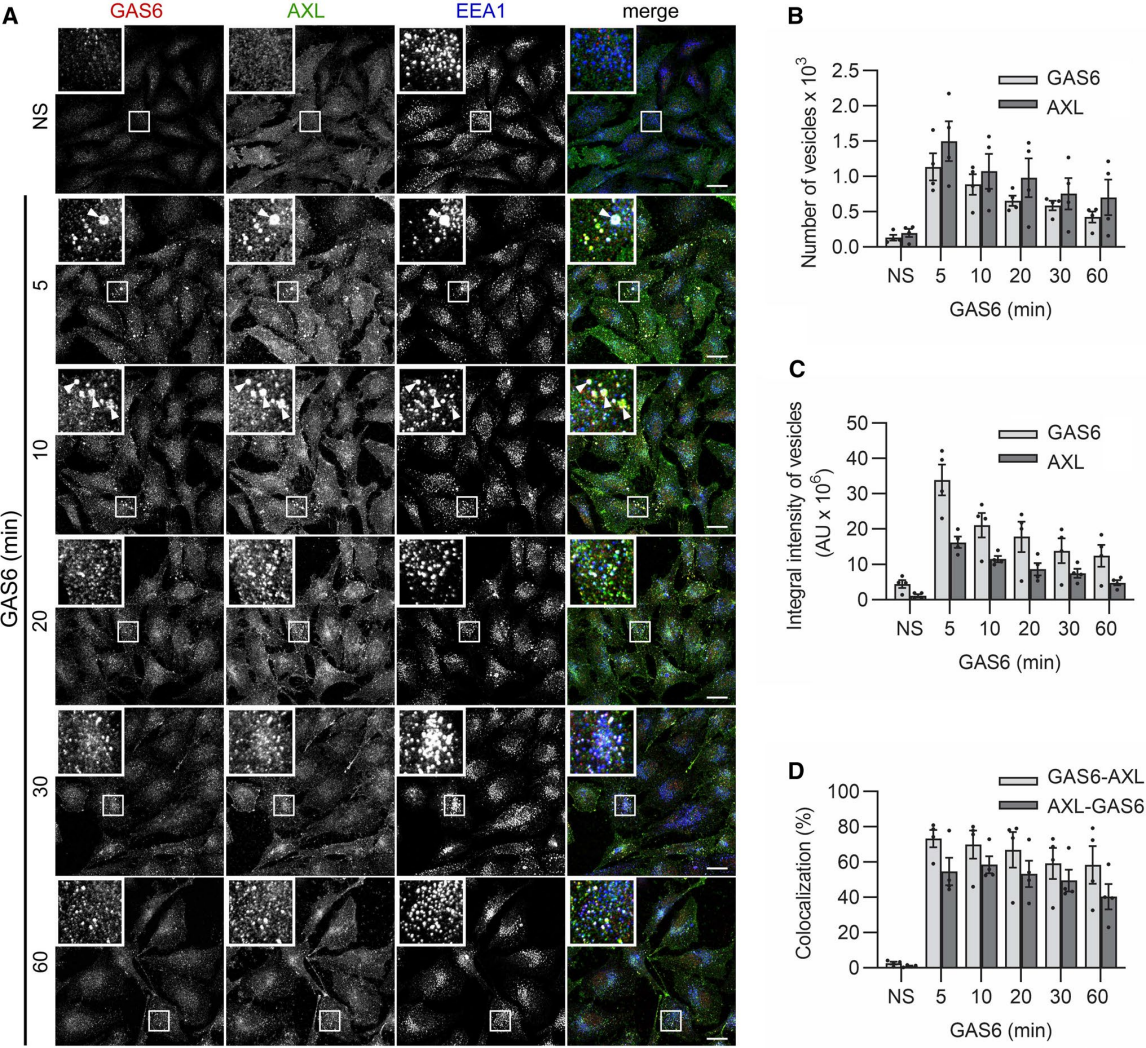
AXL and GAS6 accumulate rapidly on endosomal structures. **A** Confocal images showing the kinetics of internalization of GAS6 and AXL in LN229 cells after continuous stimulation with GAS6. Serum-starved cells were stimulated with GAS6 for the indicated time periods. Insets show magnified views of boxed regions in the main images. Scale bars: 20 μm. Arrowheads in panels of 5 and 10 min indicate macropinosomes. **B**, **C**, **D** Quantification of number (**B**), integral fluorescence intensity (**C**), and colocalization (**D**) between GAS6- and AXL-positive vesicles (representative confocal images shown in (**A**), *n* = 4. GAS6–AXL- percentage of GAS6-positive vesicles overlapping with AXL-positive vesicles, AXL-GAS6- percentage of AXL-positive vesicles overlapping with GAS6-positive vesicles. Data information: For data quantification, approximately 150 cells were analyzed per experiment. Each dot represents data from one independent experiment, whereas bars represent the means ± SEM from n experiments. *NS* non-stimulated cells, *GAS6* GAS6-stimulated cells, *AU* arbitrary units

To exclude any non-physiological effects of the pulse-chase stimulation (which includes cell incubation on ice), we performed experiments in which serum-starved LN229 cells were incubated with GAS6 at 37 °C for increasing time periods (continuous stimulation). The kinetics of AXL internalization was similar to the one obtained after pulse-chase stimulation. The highest endosomal accumulation of AXL was already observed after 5 min of GAS6 stimulation and declined in later time periods (Fig. 2A–C). However, in contrast to the pulse-chase stimulation, GAS6 and AXL resided longer on vesicular structures (Fig. 2A–C). This observation suggests that ligand–receptor complexes are continuously internalized when GAS6 is constantly present in the medium. In addition, the majority of endosomes contained both the ligand and the receptor, which was manifested by high GAS6–AXL and AXL-GAS6 colocalization (Fig. 2D). Similar results were obtained in SKOV3 cells (Fig. S5A–D).

Cumulatively, we showed that independently of the type of stimulation (pulse-chase or continuous), GAS6–AXL complexes rapidly accumulate on endosomal structures after internalization, but then the number of AXL- and GAS6-positive vesicles decreases.

### AXL colocalizes with clathrin light chain (CLC) and PICALM; however, depletion of CME regulators does not decrease GAS6–AXL endocytosis

**Fig. 3:**
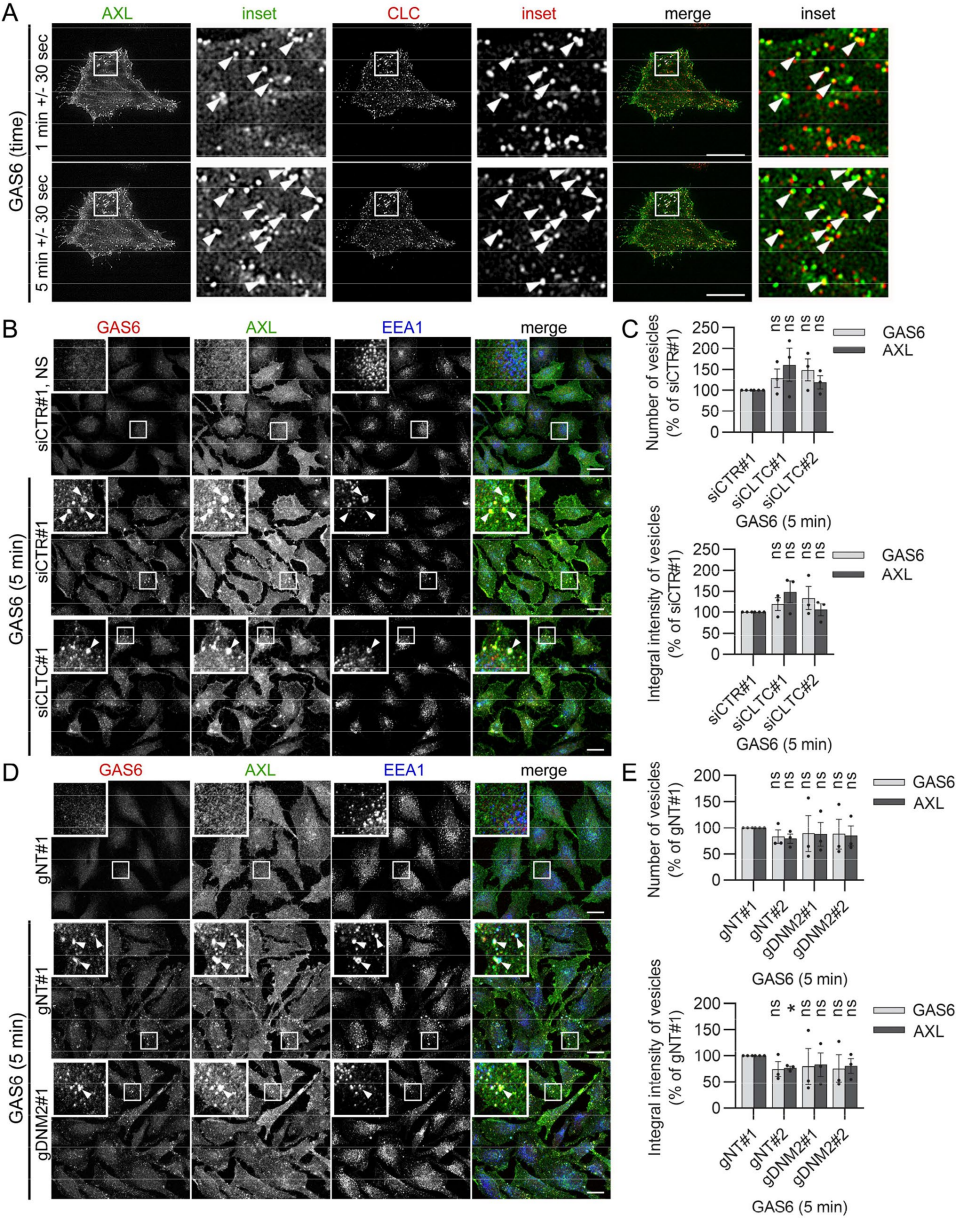
CME may contribute to GAS6–AXL internalization. **A** Total internal reflection fluorescence (TIRF) analysis of living LN229 cells expressing AXL-EGFP and mRFP-CLC. Serum-starved cells were imaged every 20 s up to 10 min after GAS6 addition. Representative frames from Movie S1 are shown. Arrowheads indicate structures positive for both AXL and CLC. **B** Confocal images showing GAS6–AXL internalization upon depletion of CHC in LN229 cells. Two siRNAs targeting *CLTC* (siCLTC#1 and siCLTC#2) were used. LN229 cells transfected with non-targeting siRNA (siCTR#1) served as control. Cells were transfected twice with 72 h break in between. 72 h after the second transfection, serum-starved LN229 cells were stimulated with GAS6 for 5 min. Arrowheads in images of GAS6-stimulated cells indicate macropinosomes. **C** Quantification of number and integral fluorescence intensity of GAS6- and AXL-positive vesicles in LN229 cells upon depletion of CHC (representative confocal images shown in (**B**), *n* = 3. Student’s one-sample *t* test, *ns* non-significant (*p* > 0.05). **D** Confocal images showing GAS6–AXL internalization upon knockout of *DNM2* in LN229 cells. Two gRNAs targeting *DNM2* (gDNM2#1 and gDNM2#2) were used. CRISPR-Cas9-edited LN229 cells with two non-targeting gRNAs (gNT#1 and gNT#2) served as controls. Serum-starved cells were stimulated with GAS6 for 5 min. Arrowheads in images of GAS6-stimulated cells indicate macropinosomes. **E** Quantification of number and integral fluorescence intensity of GAS6- and AXL-positive vesicles in CRISPR-Cas9-mediated knockouts of *DNM2* (representative confocal images shown in (**D**), *n* = 3. Student’s one-sample *t* test, *p ≤ 0.05, *ns* non-significant (*p* > 0.05). Data information: Insets in confocal images show magnified views of boxed regions in the main images. Scale bars: 20 μm. For data quantification, approximately 150 cells were analyzed per experiment. Each dot represents data from one independent experiment, whereas bars represent the means ± SEM from n experiments. *NS* non-stimulated cells, *GAS6* GAS6-stimulated cells

Our BioID data indicated that GAS6–AXL complexes might be internalized via CME as there were many proteins implicated in CME among AXL proximity interactors (Table 1, [31]). Thus, to verify this supposition, we tested the colocalization of AXL with clathrin light chain (CLC) and PICALM, a cytoplasmic adaptor protein involved in CME that was identified in the AXL interactome [31]. To this end, we performed total internal reflection fluorescence (TIRF) analysis of living LN229 cells expressing AXL-EGFP and mRFP-CLC or mCherry-PICALM. As shown in Movie S1 and Fig. 3A, and Movie S2 and Fig. S6A, some AXL-containing vesicles colocalized with CLC- or PICALM-positive vesicles. These data suggest that a fraction of AXL enters cells via CME.

**Table 1:** AXL proximity interactors involved in endocytic trafficking

Endocytic process	AXL interactors
CME	EPS15, EPS15L1, PICALM, DLG1, NUMB, CTTN, MYO6, β-actin, KIF5B, AGFG1, HIP1, SYNJ2, dishevelled, DBNL, CIN85, lamellipodin, SCYL2, ESYT2, EPSINR
CIE	CTTN, HSPA1A, SH3RF1, CD44, CD98, β-actin, annexin A2, ITGβ1, moesin, AHNAK, GPR37, RTN4, utrophin, CAV1, FLOT1, SHIP2, ROBO1, lamellipodin, merlin, MTMR6, WAVE2, SEPT7, SEPT9
Recycling	ERBIN, SCRIB, SNAP29, EHBP1, SNX1, merlin, RABFIP5, EPS1, KIF5B, GGA3, VAPM3, EHBP1L1, ASAP1

Therefore, we next verified whether deficiency in CME regulators affected GAS6–AXL endocytosis. Among AXL proximity interactors, we previously identified proteins acting as alternative clathrin adaptors such as EPS15, EPS15L1, or NUMB (Table 1) [31, 60, 61]. As we confirmed the proximity interactions of these proteins with AXL (Fig. S6B), we thus constructed CRISPR-Cas9-mediated knockouts of genes encoding them. As shown in Fig. S6C–F, depletion of EPS15, EPS15L1, or NUMB did not affect endocytosis of GAS6–AXL complexes. Therefore, we next analyzed whether CRISPR-Cas9-mediated depletion of key regulators of CME, clathrin, or DNM2, blocked GAS6–AXL endocytosis. However, we were unable to efficiently inhibit the expression of *CLTC,* a gene encoding clathrin heavy chain (CHC), using the CRISPR-Cas9 approach, probably due to the implication of clathrin in processes essential for cell viability and survival [62]. Thus, to efficiently deplete CHC, we transfected cells with siRNAs against *CLTC* twice, with a 72 h interval in between (Fig. S7A). Quantitative analysis of microscopic data revealed that siRNA-mediated depletion of CHC did not reduce GAS6 and AXL internalization (Fig. 3B, C and Fig. S7B, D), whereas endocytosis of transferrin (Tf), a well-established cargo for CME, was significantly decreased (Fig. S7C, D). Similarly, KO of *DNM2* had no effect on endocytosis of GAS6 and AXL (Fig. 3D, E and Fig. S7E–H), whereas it blocked endocytosis of Tf (Fig. S7F, G).

Cumulatively, our data indicate that a fraction of GAS6–AXL complexes are endocytosed via CME; however, the deficiency in CME is probably compensated via other clathrin-independent endocytic routes with no net effect on GAS6–AXL uptake.

### GAS6–AXL complexes are internalized via several CIE pathways

**Fig. 4:**
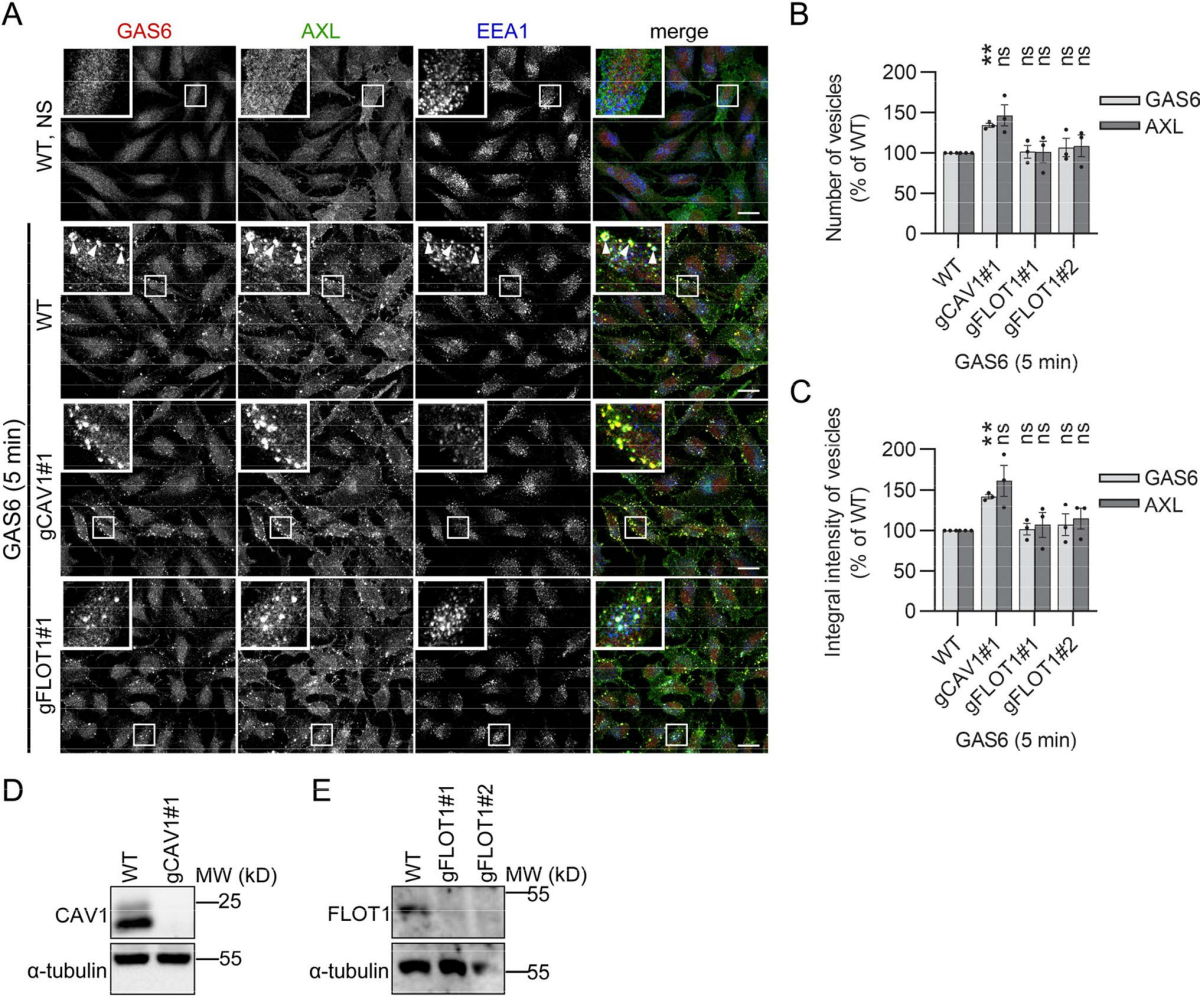
Depletion of CAV1 or FLOT1 does not decrease GAS6–AXL endocytosis. **A** Confocal images showing GAS6–AXL internalization upon CRISPR-Cas9-mediated knockout of *CAV1* or *FLOT1* in LN229 cells. Wild-type (WT) LN229 served as controls. Serum-starved cells were stimulated with GAS6 for 5 min. Arrowheads in images of WT GAS6-stimulated cells indicate macropinosomes. **B**, **C** Quantification of number (**B**) and integral fluorescence intensity (**C**) of GAS6- and AXL-positive vesicles in knockouts of *CAV1* or *FLOT1* (representative confocal images shown in (**A**), *n* = 3. Student’s one-sample *t* test, **p ≤ 0.01, *ns* non-significant (*p* > 0.05). **D**, **E** Western blots showing the efficiency of CRISPR-Cas9-mediated knockout of *CAV1* (**D**) and *FLOT1* (**E**). One gRNA targeting *CAV1* (gCAV1#1) and two gRNA targeting *FLOT1* (gFLOT1#1 and gFLOT1#2) were used. α-Tubulin served as a loading control. Data information: Insets in confocal images show magnified views of boxed regions in the main images. Scale bars: 20 μm. For data quantification, approximately 150 cells were analyzed per experiment. Each dot represents data from one independent experiment, whereas bars represent the means ± SEM from *n* experiments. *NS* non-stimulated cells, *GAS6* GAS6-stimulated cells

We previously found that AXL can be internalized by macropinocytosis induced by GAS6 stimulation [31]. In line with this, we again observed accumulation of GAS6 and AXL on macropinosomes (Figs. S1A, 2A, 3B, D, 4A). However, our AXL proximity interactome also contained proteins involved in CIE pathways other than macropinocytosis (Table 1). Thus, to verify the involvement of these pathways in GAS6–AXL internalization, we first tested endosomal accumulation of both the ligand and the receptor upon CRISPR-Cas9-mediated depletion of caveolin 1 (CAV1) or flotilin 1 (FLOT1). These two important regulators of caveolae- and flotilin-mediated endocytosis, respectively [46], were identified in our BioID data (Table1, [31]). As shown in Fig. 4A–E, depletion of neither CAV1 nor FLOT1 inhibited the uptake of GAS6–AXL complexes. In contrast, *CAV1* KO slightly elevated the GAS6–AXL internalization (Fig. 4A–C).

**Fig. 5:**
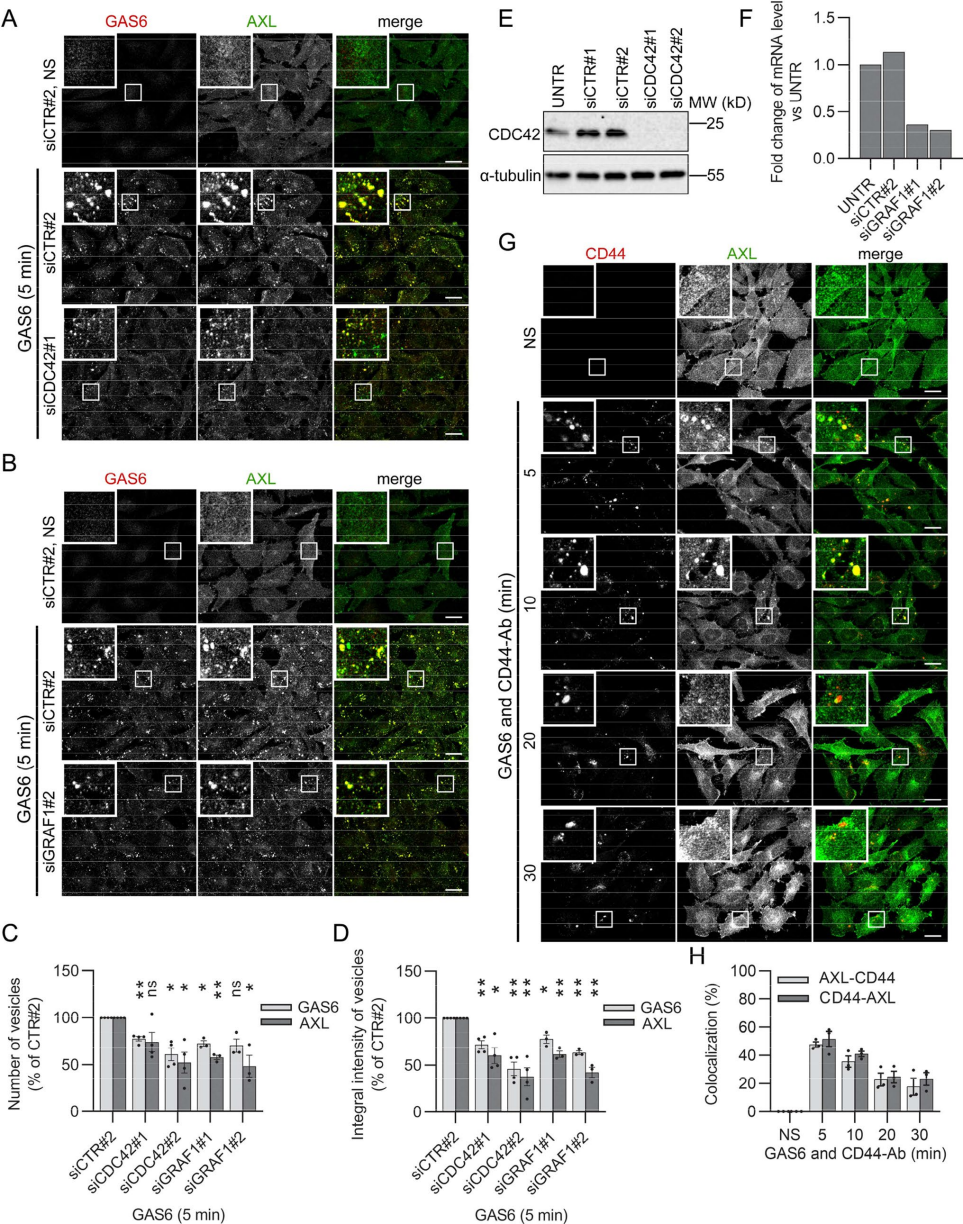
The CLIC–GEEC pathway mediates GAS6-induced AXL endocytosis. **A**, **B** Representative confocal images showing GAS6–AXL internalization upon knockdown of *CDC42* (**A**) or *GRAF1* (**B**) in LN229 cells. Two siRNAs targeting *CDC42* (siCDC42#1 and siCDC42#2) or *GRAF1* (siGRAF1#1 and siGRAF#2) were used. LN229 cells transfected with non-targeting siRNAs (siCTR#2) served as control. 72 h after transfection, serum-starved cells were stimulated with GAS6 for 5 min. **C**, **D** Quantification of number (**C**) and integral fluorescence intensity (**D**) of GAS6- and AXL-positive vesicles in cells depleted of CDC42 *n* = 4 or GRAF1 *n* = 3 (representative confocal images shown in **A** and **B**). Student’s one-sample *t* test, *p ≤ 0.05, **p ≤ 0.01, *ns* non-significant (*p* > 0.05). **E** Western blot showing efficiency of *CDC42* silencing. LN229 cells were transfected as described in A or left non-transfected (UNTR). α-Tubulin served as a loading control. **F** Graph showing silencing efficiency of *GRAF1*. LN229 cells were transfected as described in B and analyzed by qRT-PCR. Values are presented as a fold change of *GRAF1* mRNA level versus non-transfected cells (UNTR) set as 1. **G** Confocal images showing the internalization of CD44 and AXL. Serum-starved LN229 cells were stimulated with GAS6 and agonistic antibody recognizing CD44 (CD44-Ab) for the indicated time periods. **H** Quantification of colocalization between AXL- and CD44-Ab-positive vesicles (representative confocal images shown in (**G**), *n* = 3. AXL-CD44- percentage of AXL-positive vesicles overlapping with CD44-positive vesicles, CD44-AXL- percentage of CD44-positive vesicles overlapping with AXL-positive vesicles. Data information: Insets in confocal images show magnified views of boxed regions in the main images. Scale bars: 20 μm. For data quantification, approximately 150 cells were analyzed per experiment. Each dot represents data from one independent experiment, whereas bars represent the means ± SEM from *n* experiments. *NS* non-stimulated cells, *GAS6* GAS6-stimulated cells, *GAS6 and CD44-Ab* cells stimulated with GAS6 and CD44-Ab

Additionally, among AXL interactors, we found several proteins, such as CD44, β-actin, annexin A2, ITGβ1, moesin, AHNAK, GPR37, or RTN4 ((Table 1, [31]), which were previously isolated from a CLIC-enriched fraction [63]. Thus, we next assessed the effect of perturbation of the CLIC/GEEC pathway on internalization of GAS6 and AXL. As shown in Fig. 5A–F, depletion of key regulators of this pathway, CDC42 and GRAF1 [46, 56, 64, 65], partially decreased endocytosis of GAS6–AXL complexes. Similarly, internalization of GAS6 was impaired in SKOV3 cells depleted of CDC42 and GRAF1 (Fig. S8A-C). Since CD44, a well-known cargo of CLIC/GEEC, was present among AXL proximity interactors, we measured colocalization between internalized CD44 and AXL. We could reliably measure CD44 endocytosis only in cells stimulated with GAS6, which argues that GAS6 induces uptake of CD44. As shown in Fig. 5G, H, there was up to 50% colocalization between CD44- and AXL-positive vesicles upon co-stimulation with two ligands. As expected, depletion of CDC42 decreased GAS6-induced uptake of CD44 (Fig. S9A and B). These data support the notion that CD44 traffics together with AXL, and that subpopulation of AXL molecules is internalized via the CLIC/GEEC pathway (Fig. 5 and S9).

Cumulatively, our previous and present data suggest that several CIE pathways, such as macropinocytosis or CLIC-GEEC, mediate the uptake of GAS6–AXL complexes.

### AXL displays distinct kinetics of endocytosis in comparison to other RTKs

The lack of a significant impact of the perturbation of several endocytic pathways (Figs. 3, 4, 5) together with the observed rapid accumulation of GAS6 and AXL on endosomes (Fig. 2) indicate that endocytosis of AXL displays distinct features in comparison to endocytic trafficking of other RTKs. Therefore, we compared endocytosis of AXL to endocytosis of EGFR, the best-characterized RTK with respect to endocytic trafficking. Of note, EGFR was present among proximity interactors of AXL and a functional interplay between AXL and EGFR has been reported in the literature [31, 66–68].

**Fig. 6:**
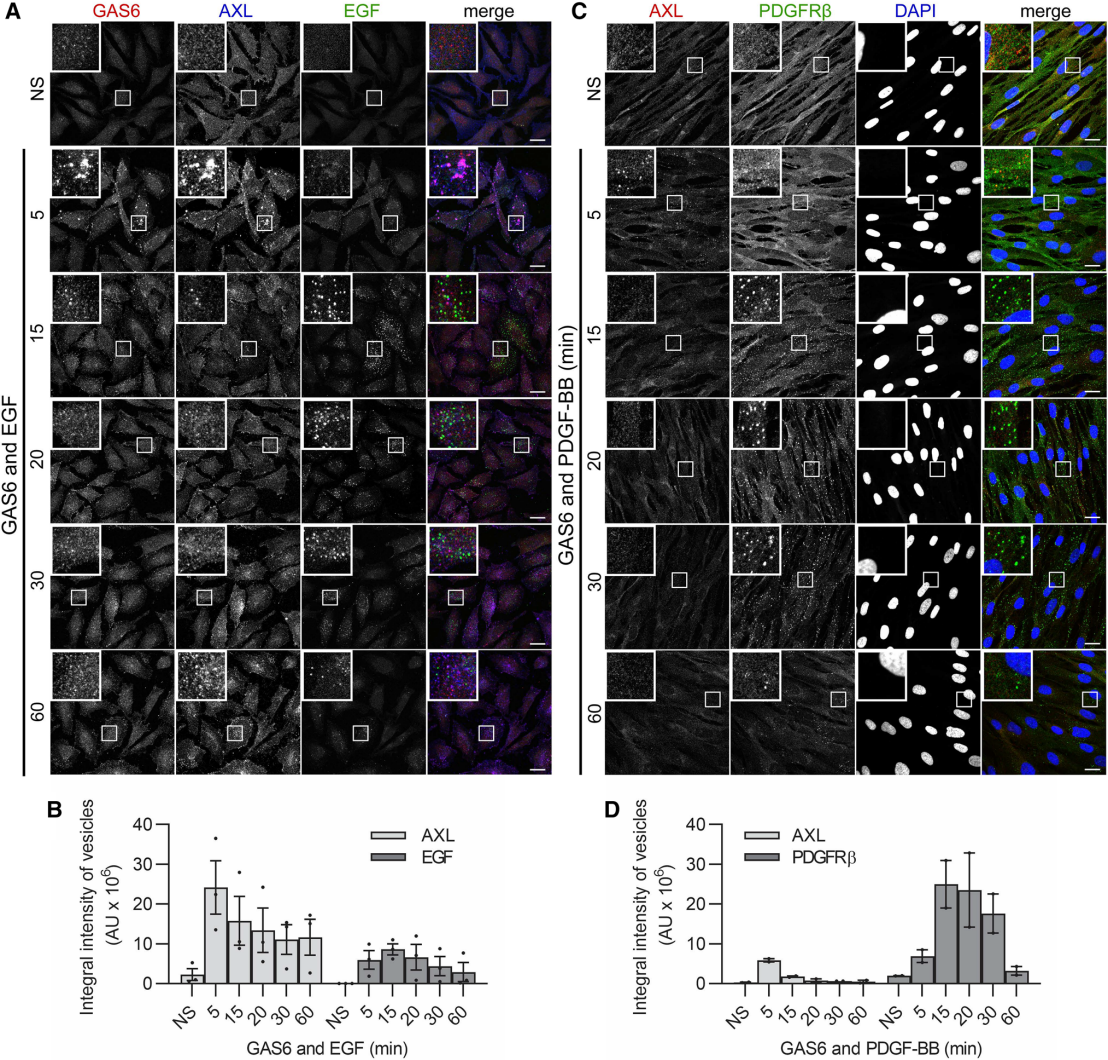
AXL displays distinct kinetics of internalization in comparison to EGFR and PDGFRβ. **A** Confocal images showing the kinetics of internalization of EGF and AXL in LN229 cells. Serum-starved cells were stimulated with EGF and GAS6 for the indicated time periods. **B** Quantification of integral fluorescence intensity of AXL- and EGF-positive vesicles (representative confocal images shown in (**A**), *n* = 3. **C** Confocal images showing the kinetics of internalization of AXL and PDGFRβ in CCD-1070Sk cells. Serum-starved cells were incubated on ice with GAS6 and PDGF-BB and after removing of unbound ligands, endocytosis was allowed to proceed at 37 °C for the indicated time periods. **D** Quantification of integral fluorescence intensity of AXL- and PDGFRβ-positive vesicles (representative confocal images shown in (**C**), *n* = 2. Data information: Insets in confocal images show magnified views of boxed regions in the main images. Scale bars: 20 μm. For data quantification, approximately 150 cells were analyzed per experiment. Each dot represents data from one independent experiment, whereas bars represent the means ± SEM from *n* experiments. *N*S non-stimulated cells, *GAS6 and EGF* cells stimulated with GAS6 and EGF, and *GAS6 and PDGF-BB* cells stimulated with GAS6 and PDGF-BB, *AU* arbitrary units

First, we assessed the kinetics of endocytosis of AXL and EGFR. To this end, we measured endosomal accumulation of epidermal growth factor (EGF), a well-studied ligand for EGFR. We found that, in contrast to GAS6–AXL complexes reaching maximal internalization at 5 min, accumulation of EGF on endosomes peaked at 10–15 min and then slowly dropped both in cells stimulated with EGF only (Fig. S10A–C) and co-stimulated with EGF and GAS6 (Fig. 6A, B). Similarly, endocytosis of AXL was faster than that of platelet-derived growth factor receptor β (PDGFRβ) in human fibroblasts CCD-1070Sk (Fig. 6C, D). Thus, these data indicate that, in comparison to other RTKs, AXL displays faster kinetics of endocytosis in cancer cells as well as in normal human fibroblasts.

**Fig. 7:**
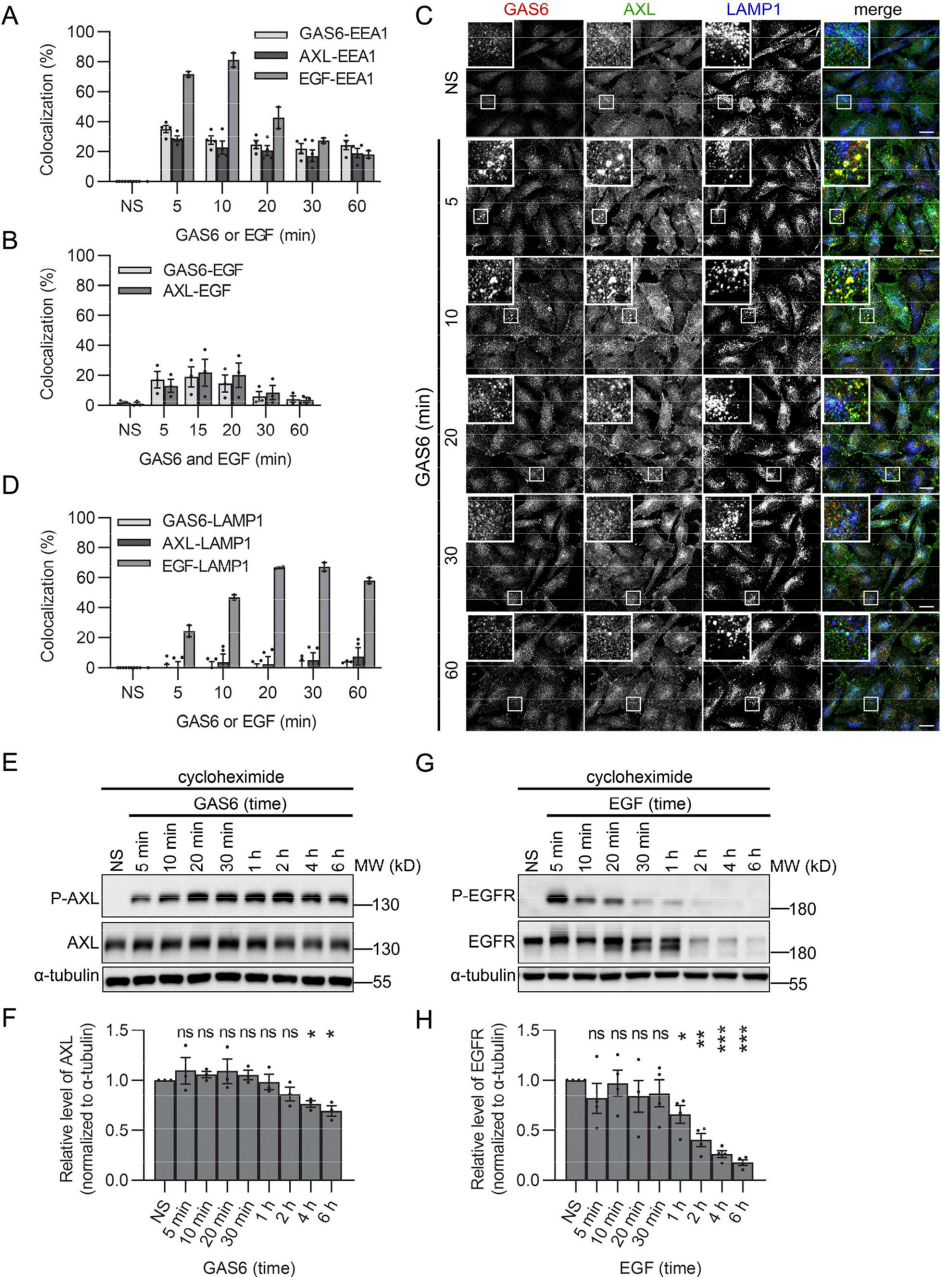
Internalized AXL is not sorted to degradation and displays prolonged signaling in comparison to EGFR. **A** Quantification of the colocalization between GAS6-, AXL- (*n* = 4) or EGF- (*n* = 2) positive vesicles with EEA1 in LN229 cells. Serum-starved cells were stimulated for the indicated time periods with GAS6 or EGF (representative confocal images are shown in Fig. 2A and Fig. S10A, respectively). **B** Quantification of the colocalization between GAS6-EGF and AXL-EGF vesicles in cells treated with both GAS6 and EGF (representative confocal images shown in Fig. 6A), *n* = 3. **C** Confocal images showing the internalization of GAS6 and AXL and their colocalization with LAMP1 in LN229 cells. Serum-starved cells were stimulated with GAS6 for the indicated time periods. **D** Quantification of the colocalization between GAS6-, AXL- (*n* = 4) or EGF- (*n* = 2) positive vesicles with LAMP1. Representative confocal images are shown in C for cells stimulated with GAS6 and in Fig. S10A for cells stimulated with EGF. **E** Western blot showing GAS6-induced phosphorylation of AXL (P-AXL, Y702) and total level of AXL after continuous stimulation with GAS6. Serum-starved LN229 cells were pre-treated with cycloheximide and stimulated with GAS6 for the indicated time periods, *n* = 3. α-Tubulin served as a loading control. **F** Graph showing the densitometric analysis of AXL levels shown in (**E**), normalized to α-tubulin, n = 3. Student’s one-sample *t* test, *p ≤ 0.05, *ns* non-significant (*p* > 0.05). **G** Western blot showing EGF-induced phosphorylation of EGFR (P-EGFR, Y1173) and total level of EGFR after continuous stimulation with EGF. Serum-starved LN229 cells were pre-treated with cycloheximide and stimulated with EGF for the indicated time periods, *n* = 4. α-Tubulin served as a loading control. **H** Graph showing the densitometric analysis of EGFR levels shown in G, normalized to α-tubulin, *n* = 4. Student’s one-sample *t* test,*p ≤ 0.05, **p ≤ 0.01, ***p ≤ 0.001, *ns* non-significant (*p* > 0.05). Data information: For data quantification approximately 150 cells were analyzed per experiment. Each dot represents data from one independent experiment, whereas bars represent the means ± SEM from *n* experiments. *NS* non-stimulated cells, *GAS6* GAS6-stimulated cells, *EGF* EGF-stimulated cells, *GAS6 and EGF* GAS6- and EGF-stimulated cells, *GAS6 or EGF* GAS6- or EGF-stimulated cells

Next, to identify the population of endosomes through which GAS6–AXL complexes and EGF traffic, we measured the colocalization of GAS6-, AXL-, or EGF-positive vesicles with EEA1. As shown in Figs. 2A, 7A, the majority of GAS6–AXL complexes did not traffic through EEA1-positive vesicles. In contrast, EGF displayed up to 80% colocalization with EEA1 after 10 min of stimulation (Fig. 7A, Fig. S10A). Moreover, we also observed a limited colocalization between AXL and EGF vesicles in LN229 cells (Figs. 6A, 7B).

Taken together, our results showed that AXL displays faster kinetics of internalization in comparison to other RTKs, such as EGFR and PDGFRβ. Additionally, the limited colocalization of AXL with EEA1 and EGF indicates that the majority of AXL molecules traffic through different endosomal compartments than EGFR.

### Decrease in endosomal accumulation of AXL does not result from its degradation but rather from its recycling via SNX1-positive endosomes

It is known that upon internalization, receptors reach early endosomes from which they are targeted for degradation, via late endosomes and lysosomes, or are recycled back to the plasma membrane [69]. To verify whether AXL enters the degradative endo-lysosomal compartment, we checked its colocalization with LAMP1, a marker of late endosomes and lysosomes. As shown in Fig. 7C, D, GAS6- and AXL-positive endosomes did not colocalize with LAMP1 even at prolonged periods of stimulation. In contrast, the colocalization of LAMP1 with ligand-bound EGFR, a well-described cargo that is sorted for degradation [70], increased in time reaching 65% at 20 min of stimulation (Fig. 7D and Fig. S10A).

To additionally confirm that AXL is not sorted to degradation, we checked its level upon GAS6 stimulation. To prevent de novo synthesis of proteins, LN229 cells were pre-incubated with cycloheximide (CHX), and next treated with GAS6 for increasing time periods. Western blot analysis showed that the level of AXL was stable up to 4 and 2 h during pulse-chase (Fig. S10D, E) and continuous (Fig. 7E, F) stimulation with GAS6, respectively. The subsequent degradation was slow and over 60% of AXL was still detectable after 6 h of both pulse-chase and continuous GAS6 stimulation. This was in contrast to EGFR that remained stable only up to 30 min, followed by its fast degradation with 18% of EGFR left after 6 h of EGF stimulation (Fig. 7G, H).

**Fig. 8:**
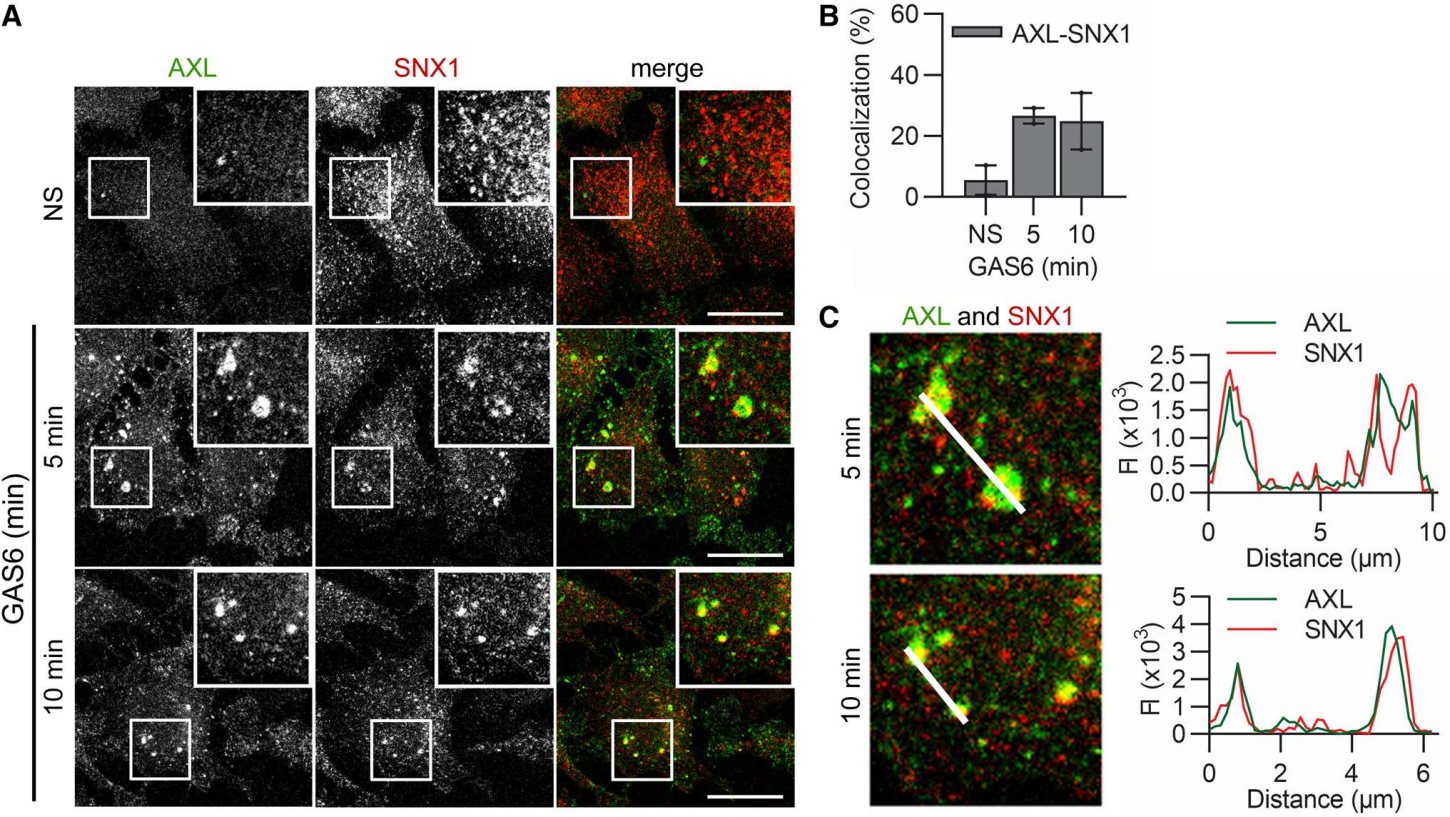
GAS6-stimulated and internalized AXL colocalizes with SNX1. **A** Confocal images showing the colocalization between AXL and SNX1 in LN229 cells. Serum-starved cells were stimulated with GAS6 for 5 and 10 min. **B** Quantification of colocalization between AXL- and SNX1-positive vesicles (representative confocal images are shown in (**A**), *n* = 2. **C** Fluorescence intensity profiles (FI) along the white lines. Insets from confocal images presented in (**A**) are shown. Data information: For data quantification, approximately 150 cells were analyzed per experiment. Each dot represents data from one independent experiment, whereas bars represent the means ± SEM from n experiments. *NS* non-stimulated cells, *GAS6* GAS6-stimulated cells

Inefficient degradation of GAS6-stimulated AXL and fast decrease in its endosomal accumulation indicate that AXL may be predominantly recycled back to the plasma membrane. This notion is supported by our previous BioID data, as multiple proteins involved in endocytic recycling were present among AXL interactors (Table 1, [31]). SNX1, an established regulator of recycling [71, 72], was one of the strongest BioID hits [31] and we confirmed its proximity interaction with AXL (Fig. S6B). Furthermore, AXL and SNX1 partially colocalized upon GAS6 stimulation (Fig. 8A–C). This suggests that a fraction of the internalized receptor is recycled via SNX1-positive endosomes.

### The kinetics of AKT activation upon GAS6 stimulation depends on endocytic trafficking of GAS6–AXL complexes

**Fig. 9:**
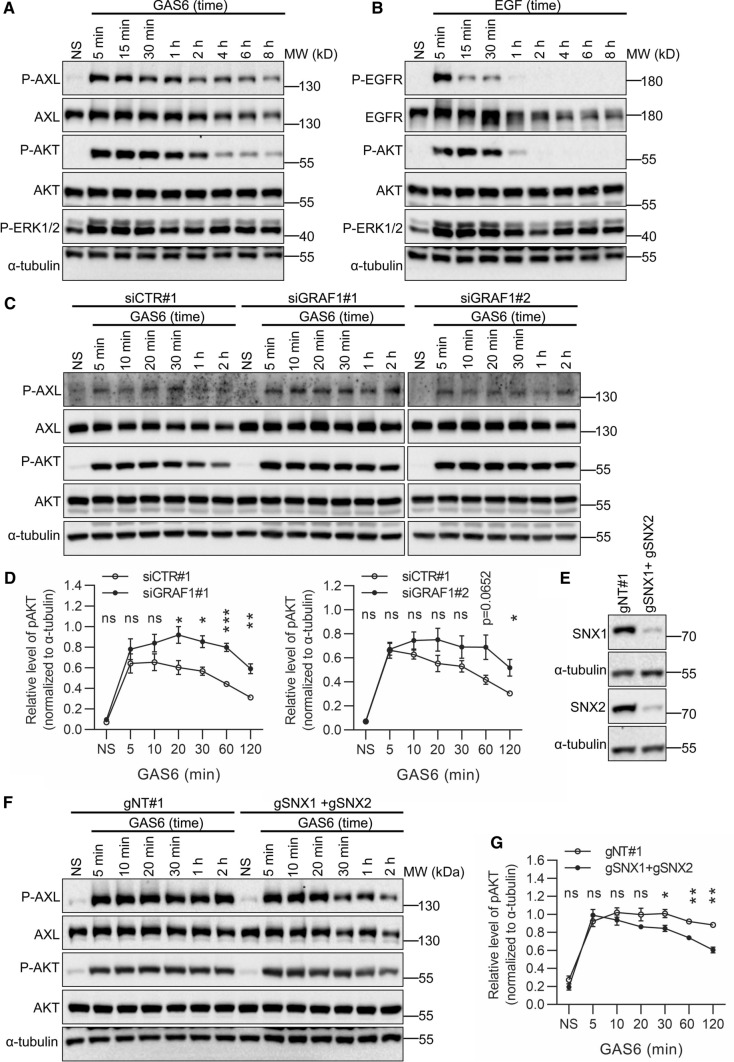
GAS6–AXL signaling causes prolonged phosphorylation of AKT. **A**, **B** Western blot showing phosphorylation of AXL (P-AXL, Y702) (**A**) and EGFR (P-EGFR, Y1173) (**B**) and their downstream effectors, AKT (P-AKT, S473) and ERK1/2 (P-ERK, T202/T204). Serum-starved LN229 cells were stimulated with GAS6 (**A**) or EGF (**B**) for increasing time periods, lysed and immunoblotted against the indicated proteins. α-Tubulin was used as a loading control. **C** Western blot showing phosphorylation of AXL (P-AXL, Y702) and its downstream effector AKT (P-AKT, S473) in LN229 cells depleted of GRAF1. Two siRNAs targeting *GRAF1* (siGRAF1#1 and siGRAF#2) were used. LN229 cells transfected with non-targeting siRNAs (siCTR#1) served as control. 72 h after transfection serum-starved cells were stimulated with GAS6 for the indicated time periods. **D** Graphs showing the densitometric analysis of P-AKT levels in LN229 cells depleted of GRAF1 (shown in **C**), normalized to α-tubulin, *n* = 3. Student’s unpaired *t* test,*p ≤ 0.05, **p ≤ 0.01, ***p ≤ 0.001, *ns* non-significant (*p* > 0.05). **E, F** Western blots showing the efficiency of CRISPR-Cas9-mediated double knockout (KO) of *SNX1* and *SNX2* (E) and phosphorylation of AXL (P-AXL, Y702) and its downstream effector AKT (P-AKT, S473) upon double knockout of *SNX1* and *SNX2* (F) in LN229 cells. One gRNA targeting *SNX1* (gSNX1) and one gRNA targeting *SNX2* (gSNX2) were used, along with non-targeting gRNA (gNT#1) in control cells. In F, serum-starved cells were stimulated with GAS6 for the indicated time periods. α-Tubulin served as a loading control. **G** Graphs showing the densitometric analysis of P-AKT levels in SNX1 and SNX2 KO LN229 cells (shown in **F**), normalized to α-tubulin, *n* = 3. Student’s unpaired *t* test,*p ≤ 0.05, **p ≤ 0.01, *ns* non-significant (*p* > 0.05)

Since RTK degradation and endocytic recycling affect the duration of RTK-mediated signaling [35, 73], we tested the phosphorylation status of AXL and its downstream effectors after stimulation with GAS6, in comparison to EGF-induced EGFR activation. As shown in Fig. 9A, phosphorylation of AXL and its main downstream effector in LN229 cells [10, 31], AKT, were sustained up to 8 h of GAS6 stimulation. In contrast, phosphorylation of EGFR and AKT was detected only up to 1 h of EGF stimulation, and this reflects the fact that majority of internalized EGFR is sorted to degradation (Fig. 9B). In contrast to AKT activation displaying different kinetics for AXL and EGFR, the levels of ERK1/2 phosphorylation were similar for both receptors.

To verify whether GAS6-induced AKT activation depended on endocytic trafficking of AXL, we perturbed internalization and recycling of the receptor. GAS6-stimulated phosphorylation of AKT was increased upon GRAF1 depletion (Fig. 9C, D) that also led to impairment of AXL uptake (Fig. 5B–D). Conversely, the levels of AKT phosphorylation were reduced in GAS6-treated cells bearing CRISPR-Cas9-mediated knockouts of *SNX1* and *SNX2* (depletion of both proteins was reported to be necessary for efficient inhibition of recycling [71, 72]; Fig. 9E–G).

Altogether, these data confirm the coupling between AXL endocytic trafficking and AKT signaling upon GAS6 stimulation. Specifically, reduced internalization of GAS6–AXL intensifies AKT signaling, whereas impaired recycling attenuates AKT signaling. This further suggests that the plasma membrane (and not endosomes) represents the primary site of AKT activation upon GAS6 stimulation.

## Discussion

**Fig. 10:**
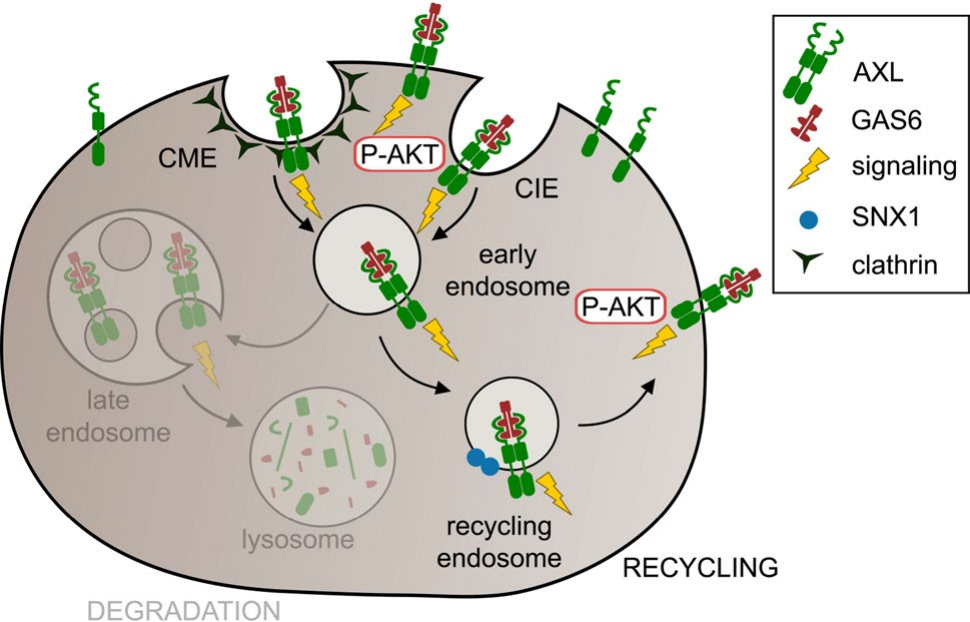
A model showing GAS6-induced AXL endocytosis and the fate of internalized AXL receptor. GAS6 stimulation triggers internalization of AXL via CME and CIE pathways. The majority of internalized AXL is not sorted toward degradation, but rather recycled back to the plasma membrane through an SNX1-dependent pathway. This results in sustained phosphorylation of AXL and its downstream effector AKT

Here, we characterized endocytic trafficking of AXL, a member of TAMs, the subfamily of receptors poorly studied in this respect [31]. We showed that upon ligation, GAS6–AXL complexes are rapidly internalized into cells via multiple endocytic pathways including both CME and CIE, and this process requires kinase activity of AXL. The observed here fast kinetics of AXL endocytosis distinguishes AXL from other RTKs, such as EGFR or PDGFRβ. Intriguingly, we found that, except for the CLIC/GEEC route, blocking a single endocytic pathway does not efficiently reduce endocytosis of GAS6–AXL complexes, indicating that other pathways compensate for the lack of one of them. Moreover, our data indicate that, in contrast to EGFR, the majority of internalized AXL is not sorted toward degradation, but at least in part recycled back to the plasma membrane via SNX1-positive endosomes (Fig. 10). This trafficking pattern is associated with prolonged duration of signaling induced by AXL, in comparison to fast-degraded EGFR. Importantly, perturbations of AXL endocytic trafficking affect the kinetics of AKT activation upon GAS6 stimulation.

Endocytosis is a major regulator of RTK function, and RTKs undergo both constitutive and ligand-induced endocytosis, which determines their half-life and signaling. To date, several RTKs were found to use multiple endocytic pathways; however, the purpose of this diversity is not entirely known. It was proposed that different endocytic routes can determine the intracellular fate of the receptor (degradation or recycling) or activate particular downstream signaling pathways and cellular responses. In turn, the choice of a specific internalization route can be regulated by ligand concentrations and/or depend on a ligand, receptor or cell type [35, 74]. In the present study, we identified the endocytic routes of ligand-bound AXL and we found that GAS6–AXL complexes enter cells through both CME and CIE. It is known that CIE pathways rely on actin polymerization [47]. Thus, the involvement of CIE in the internalization of GAS6–AXL complexes is consistent with our previous observation that GAS6-mediated AXL activation triggers actin remodeling [31]. Although CME and CIE pathways were already implicated in internalization of other RTKs, endocytosis of ligand-bound AXL displayed some unique features.

First, we observed that interfering with the components of CME or CIE did not substantially reduce AXL internalization, except for CDC42 and GRAF1 depletion, which could suggest a predominant role of the CLIC/GEEC pathway in AXL uptake. Thus, our data imply that, in contrast to EGFR or PDGFRβ, the entry routes of AXL are largely interchangeable, at least in terms of the total amount of the internalized receptor [75, 76]. However, we cannot exclude that uptake of GAS6–AXL complexes via a particular endocytic pathway is required for triggering specific cellular response. Strikingly, blocking CME via efficient CHC depletion did not reduce internalization of GAS6–AXL complexes, although AXL-positive vesicles colocalized with clathrin-coated vesicles. Thus, our results indicate that colocalization studies might constitute a better approach to analyze the involvement of particular endocytic routes in RTK endocytosis than downregulation of endocytic regulators.

Second, the overall internalization of AXL is faster than endocytosis of other RTKs such as EGFR or PDGFRβ. This indicates that in case of AXL, the predominant pathways responsible for the uptake of this receptor are fast clathrin-independent endocytic processes like macropinocytosis (our prior and present study), CLIC/GEEC (this study), and possibly FEME (several molecular players implicated in FEME, e.g., PI3K, SHIP2, lamellipodin, and ROBO1 are present among proximity interactors of AXL) (Table 1) [31, 63, 77–79]. This can be particularly important during cell spreading, migration, and invasion, when receptors need to be rapidly removed from one region of the plasma membrane, and delivered through endocytic recycling to another region [80, 81]. Particularly, both CLIC*/*GEEC and FEME were shown to operate at the leading edges of migrating cells, and we and others found that AXL is also enriched in this region [31, 82]. In line with this, our prior study demonstrated that activation of GAS6–AXL signaling enhanced spreading and invasion of cancer cells, and macropinocytosis contributed to these processes [31]. Importantly, we and others revealed that GAS6–AXL-induced macropinocytic uptake of albumin and cell debris improves the survival of cancer cells under nutrient-deprived conditions [31, 83]. Moreover, it was proposed that signaling triggered by some receptors needs to be tightly regulated to avoid overstimulation, and fast endocytosis pathways like macropinocytosis and CLIC*/*GEEC that internalize large membrane areas along with many receptor molecules prevent excessive signaling [63, 77]. Consistent with this notion, it is also possible that fast AXL internalization might protect cells from overstimulation.

Third, in comparison to EGFR, AXL traffics mostly through a different population of endosomes as AXL-positive vesicles displayed limited colocalization with vesicles containing EGFR and early endosomes marked with EEA1. It is known that internalized receptors can traffic through different endosomes toward degradation or recycling, facing distinct molecular environments on endosomal membranes and in various intracellular regions (peripheral or perinuclear endosomes) [84, 85]. For example, it was demonstrated that peripherally located APPL1 endosomes are responsible for fast recycling of EGFR and β1 integrins, increased focal adhesion turnover, and enhanced migratory and invasive phenotypes of cancer cells expressing mutated p53 [86]. Taking into account that AXL also triggers a similar migratory and invasive program and probably undergoes quick recycling, it is plausible that AXL might traffic through APPL1-positive compartments; however, this requires further investigation.

Our study also shows that GAS6 and AXL do not accumulate on LAMP1-positive vesicles. Consequently, we did not observe substantial AXL degradation upon GAS6 stimulation. Instead, internalized AXL colocalizes with vesicles positive for SNX1, a regulator of recycling and AXL proximity interactor. This, together with the presence of further proteins implicated in recycling among AXL interactors, suggests that upon internalization, GAS6–AXL complexes are recycled back to the plasma membrane. Our data also indicate that this redelivery of AXL to the plasma membrane might be specifically responsible for the sustained activation of AKT to promote cancer-cell migration and invasion [86–88], especially that the plasma membrane is a major site of activation of this effector kinase [89, 90]. Moreover, we showed that a substantial fraction of internalized AXL stays bound to GAS6, and thus, most likely AXL is redelivered to the plasma membrane in an activated, ligand-bound state. Studies of Abu-Thuraia et al*.* and us showed that activation of GAS6–AXL signaling triggers focal adhesion turnover, with AXL being localized in the close proximity to focal adhesions [31, 34]. It is thus plausible that rapid uptake of AXL and its subsequent recycling and delivery to nascent focal adhesions might play an important role during GAS6–AXL-induced cancer-cell migration and invasion. However, the exact mechanisms that may link AXL internalization or recycling with cellular responses will be difficult to address. Our results showed that perturbation of a single endocytic pathway has only minor, if any, effect on overall GAS6–AXL internalization, suggesting the involvement of several endocytic pathways in this process. Moreover, important molecular players of a given endocytic or recycling pathway have often pleiotropic cellular functions, which hinders drawing conclusions about direct links between endocytic events and a given cellular response.

Multiple studies have already demonstrated that the induction of intrinsic tyrosine kinase activity of EGFR is required for its endocytic uptake [91–97]. However, some reports argue against the involvement of the EGFR kinase activity in the internalization of this receptor [98, 99]. Here, we showed that the kinase activity of AXL was required for its internalization. Moreover, we discovered that depletion of AXL was sufficient for a complete inhibition of endosomal accumulation of GAS6 both in AXL- and TYRO3-expressing LN229 cells as well as in SKOV3 cells, which express all three TAMs [59]. In line with this, we previously showed that depletion of AXL but not TYRO3 blocked GAS6-induced processes such as membrane ruffling, macropinocytosis, and invasion [31]. Thus, our previous and current studies question a long-standing consideration in the TAM receptor field that GAS6 activates all three TAMs, and provide evidence that AXL is the primary receptor for GAS6 [5, 31, 100–103]. This further implies that the other TAM receptors do not compete with AXL for GAS6 binding.

A detailed characterization of AXL endocytosis is of a particular importance as this process is used by some viruses to gain access to cells, and AXL has been proposed to be an entry receptor for several viruses, including ZIKV and SARS-CoV-2 [24, 28, 104, 105]. Early reports indicated that AXL specifically increases entry of Lassa and Ebola viruses through enhancing macropinocytosis [22, 23]. Consistent with this, our prior and current study revealed that GAS6-mediated activation of AXL induces macropinocytosis [31]. In contrast to Lassa and Ebola viruses, AXL-mediated ZIKV entry was shown to be dependent on CME [24, 106]. Altogether, AXL-dependent virus uptake seems to rely on different endocytosis routes. Here, we discovered that GAS6–AXL complexes are endocytosed via several endocytic pathways and perturbation of one of them does not completely inhibit the ligand-receptor internalization. In turn, the inhibition of AXL kinase activity abolished GAS6-mediated uptake of AXL entirely. This indicates that inhibition of AXL, but not targeting a single endocytic pathway, may constitute a better therapeutic strategy for an antiviral treatment. In line with this, AXL-dependent cell entry of ZIKV, SARS-CoV-2, Lassa, and Ebola viruses has been previously shown to require the kinase activity of AXL [22–24, 28]. Notably, one of the AXL inhibitors, R428 (bemcentinib), is currently being tested in a clinical trial in COVID-19 patients [26, 29, 30]. Given this, our data provide a mechanistic explanation for previous virology reports showing decreased viral infection in cells treated with AXL inhibitors, and offer a rationale for pharmacological inhibition of AXL in antiviral therapy.

In summary, we discovered that endocytosis of GAS6–AXL complexes is a rapid process mediated by both CME and CIE. The majority of the internalized AXL is not degraded but recycled to specifically sustain the downstream activation of AKT at the plasma membrane, that may promote GAS6–AXL-induced cancer-cell migration and invasion. Altogether, our study uncovers molecular mechanisms of a previously uncharacterized GAS6-mediated AXL trafficking and the links between AXL endocytosis and signaling.

## Materials and methods

### Statistical methods

Data are provided as means ± SEM from at least three independent experiments, unless stated otherwise. Statistical analysis was performed using Student's one-sample t test or Student's unpaired t test using GraphPad Prism version 9. The significance of mean comparison is annotated as follows: ns, non-significant (*p*>0.05), *p≤0.05, **p≤0.01, ***p≤0.001, and ****p≤0.0001.

### Reagents

#### Inhibitors

Cycloheximide (8682.1, used at 10 µg/mL) from Carl Roth GmbH, AXL inhibitors: R428 (HY-15150, used at 5 µM) and LDC1267 (HY-12494, used at 5 µM) from MedChemExpress.

#### Others

Transferrin–Alexa-Fluor-647 (#T23366 used at 25 μg/mL), EGF-Alexa-Fluor-555 (E35350, used at 400 ng/mL), vitamin K1 (3804.1, Carl Roth GmbH), puromycin (Toku-E, P001, used at 1 µg/mL), Geneticin® Selective Antibiotic (G418, 11,811,031, used at 1 mg/mL) all from Thermo Fisher Scientific; DAPI (D9542), phalloidin-Atto 390 (50,556) both from Sigma-Aldrich; EGF (AF-100–15, used at 400 ng/mL), PDGF-BB (100-14B, used at 50 ng/mL) both from PeproTech.

### Antibodies

#### Primary antibodies

Goat anti-AXL (sc-1096, immunofluorescence (IF) 1:400), rabbit anti-AXL (sc-20741, Western blot (WB) 1:1000, IF 1:200—Figs. 5G, 8A), mouse anti-c-Myc (sc-40, IF 1:500), rabbit anti-PDGFRβ (sc-432, WB 1:1000, IF 1:200), rabbit anti-CDC42 (sc-87, WB 1:1000), mouse anti-EPS15 (sc-390259, WB 1:1000), mouse anti-flotillin 1 (sc-74566, WB 1:1000), mouse anti-DNM2 (sc-166525, WB 1:5000) all from Santa Cruz Biotechnology; goat anti-AXL (AF154, WB 1:1000) from R&D Systems; mouse anti-AKT (2920, WB 1:2000), rabbit anti-phospho-AKT (Ser 473) (4060, WB 1:1000), mouse anti-ERK1/2 (p44/42 MAPK, 9107, WB 1:1000), rabbit anti-phospho-ERK1/2 (p44/42 MAPK; Thr202/Tyr204, 4370, WB 1:1000), rabbit anti-c-Myc (2272S, IF 1:200—Figs. S8A, S9A), rabbit anti-TYRO3 (5585, WB 1:1000), rabbit anti-phospho-AXL (Tyr702) (5724, WB 1:1000), rabbit anti-NUMB (2756, WB 1:1000) all from Cell Signaling Technology; mouse anti-α-tubulin (T5168, WB 1:10,000), mouse anti-β-actin (A5441, WB 1:5000) all from Sigma-Aldrich; rabbit anti-EEA1 (ALX-210–239, IF 1:1000) from Enzo Life Sciences; mouse anti-clathrin heavy chain (CHC, 610,499, WB 1:5000), mouse anti-phospho-EGFR (Tyr1170) (558,382, WB 1:1000), mouse anti-SNX1 (611,482, IF 1:200) all from BD Biosciences; rabbit anti-EPS15L1 (ab76004, WB 1:1000), rabbit anti-EGFR (ab52894, WB 1:1000) both from Abcam; rabbit anti-caveolin (PA1-064, WB 1:1000) from Thermo Fisher Scientific; mouse anti-CD44 agonistic antibody (338,802, antibody internalization assay 1:100) from BioLegend.

#### Secondary antibodies used for WB

Horseradish peroxidase (HRP)-conjugated anti-mouse-IgG (111-035-062), anti-rabbit-IgG (111-035-144) and anti-goat-IgG (805-035-180) antibodies from Jackson ImmunoResearch; anti-rabbit-IgG conjugated to IRDye 680 (926-68,023), anti-goat-IgG conjugated to IRDye 800CW (926-32,214), and anti-mouse-IgG conjugated to IRDye 800CW (926-32,212) antibodies used in the Odyssey system were from LICOR Biosciences.

#### Secondary antibodies used for IF

Alexa Fluor 488-, 555-, 647-conjugated anti-goat-IgG, anti-mouse-IgG, and anti-rabbit-IgG were from Thermo Fisher Scientific.

### Plasmids

pmRFP-CLC and pmCherry-N1-PICALM were kindly provided by K.O. Schink (Institute for Cancer Research, Oslo University Hospital, Oslo, Norway). pEGFP-N2-AXL plasmid was constructed as described elsewhere [31]. LentiCRISPRv2 was a gift from Feng Zhang (Addgene plasmid #52,961; http://n2t.net/addgene:52961; RRID:Addgene_52961). Lentiviral packaging plasmids: psPAX2 (a gift from Didier Trono, Addgene plasmid #12,260; http://n2t.net/addgene:12260; RRID:Addgene_12260) and pMD2.G (a gift from Didier Trono, Addgene plasmid #12,259; http://n2t.net/addgene:12259; RRID:Addgene_12259). gRNA sequences for CRISPR-Cas9-mediated *DNM2*, *EPS15*, *EPS15L1*, *NUMB*, *CAV1*, and *FLOT1* inactivation (Table S1) were cloned into the LentiCRISPRv2 vectors using a protocol described elsewhere [107]. Plasmids with gRNA sequences targeting *AXL* or *TYRO3,* as well as non-targeting gRNA were generated as described before [31].

### Purification of GAS6-MycHis

GAS6-MycHis was purified as described previously [31].

### Cell culture

LN229 cells were maintained in Dulbecco’s Modified Eagle’s Medium (DMEM) high glucose, SKOV3 cells in McCoy’s 5A medium, and CCD-1070Sk cells in Minimum Essential Medium (MEM), all supplemented with 10% fetal bovine serum (FBS) and 2 mM L-glutamine (all from Sigma-Aldrich). All cell lines were purchased form ATCC. Cells were cultured at 37 °C and 5% CO_2_, and regularly tested for mycoplasma contamination.

### Cell stimulation and treatment with inhibitors

For immunofluorescence (IF) 5 × 10^4^ cells/well or 2 × 10^4^ cells/well (for siRNA transfection experiments) of LN229 cells, 4 × 10^4^ cells/well or 1.5 × 10^4^ cells/well (for siRNA transfection) of SKOV3 cells and 5 × 10^4^ cells/well of CCD-1070Sk cells were seeded on 12-mm coverslips in 24-well plates. Before stimulation, cells were incubated in serum-free medium for 16 h. On the day of stimulation, medium was exchanged to CO_2_-independent medium (Thermo Fisher Scientific) or 1 M HEPES pH 7.5 was added to the culture medium dedicated to a given cell line to final concentration of 20 mM, and cells were next incubated with 400 ng/mL GAS6-MycHis (for simplicity called GAS6), 400 ng/mL EGF-Alexa-Fluor-555, 400 ng/mL EGF, 50 ng/mL PDGF-BB, or 5 µg/mL anti-CD44 antibodies for the indicated time periods at 37 °C outside of the CO_2_ incubator.

For pulse-chase stimulation, medium was exchanged to cold CO_2_-independent medium and serum-starved cells were incubated with GAS6 on ice for 30 min to allow ligand binding. Next, cells were washed with cold CO_2_-independent medium to remove unbound ligand and incubated with warm medium for the indicated time periods at 37 °C to allow endocytosis.

For WB, 3 × 10^5^ cells/well or 1.5 × 10^5^ cells/well (for siRNA transfection) of LN229 cells were seeded in 6-well plates and stimulated as described for IF. In case of siRNA transfection experiments, cells were stimulated with GAS6 72 h after transfection, unless stated otherwise. For inhibitor treatment, cells were incubated with appropriate concentration of the indicated inhibitor for 30 min at 37 °C prior to stimulation with GAS6. In control samples, the same volume of DMSO was added.

### Immunofluorescence (IF) staining and image analysis

After stimulation, cells were washed twice with ice-cold PBS for 5 min, fixed with 3.6% paraformaldehyde in PBS for 10 min at room temperature, and stained according to the immunofluorescence protocol with saponin permeabilization, as described elsewhere [108]. Briefly, fixed cells on coverslips were permeabilized and blocked with saponin solution I (0.1% (w/v) saponin, 0.2% (w/v) fish gelatin, and 5 mg/ml BSA in PBS) for 10 min, and incubated with primary antibodies diluted in saponin solution II (0.01% (w/v) saponin, 0.2% (w/v) fish gelatin in PBS) for 1 h. Next, coverslips with cells were washed twice with saponin solution II and incubated for 30 min with secondary antibodies diluted in saponin solution II. Finally, coverslips with cells were washed three times with PBS, briefly soaked in water, and mounted on glass slides using Mowiol solution (100 mM Tris pH 8.5, 25% glycerol, and 10% polyvinyl alcohol). To visualize endocytosis of GAS6 and AXL, cells were immunostained with antibodies against Myc and AXL, respectively. Additionally, Phalloidin-Atto 390 or DAPI to stain actin or nuclei, respectively, were added during incubation with fluorescent secondary antibodies.

Twelve-bit images with resolution 1024 × 1024 pixels were acquired using the LSM 710 confocal microscope (Zeiss) with ECPlan-Neofluar 40 × 1.3 NA oil immersion objective. ZEN 2 software (Zeiss) was used for acquisition. Quantitative image analysis of endocytosis was performed using the MotionTracking software (http://motiontracking.mpi-cbg.de) [109, 110]. The first step of this analysis was the identification of objects (vesicles) in individual channels based on parameters such as size and fluorescence intensity of objects, resolution limit and noise distribution. Subsequently, a user-defined mask was applied to each image to identify any areas of the image not covered with cells and to exclude them from further analysis. Next, the program calculated the relevant parameters which included a number and integral intensity of objects (sum of fluorescence intensity coming from objects) in a particular channel (expressed in arbitrary units, AU) and colocalization between objects from two different channels (expressed in percent). Data of single experimental condition (number of vesicles, integral intensity of vesicles, or % of colocalization) are averaged from ten images (approximately 150 cells). All pictures were assembled in Photoshop (Adobe) with only linear adjustments of contrast and brightness.

### siRNA transfection

Twenty-four hours after seeding, cells were transfected with siRNA using Lipofectamine RNAiMAX (Thermo Fisher Scientific) according to the manufacturer’s protocol. The final concentration of siRNA was 10 nM. Sequences of the Ambion Silencer Select siRNAs (Thermo Fisher Scientific) used in the study are listed in Table S2. Cells were analyzed 72 h upon transfection and silencing efficiency was assessed by WB or qRT-PCR. For silencing of *CLTC*, 2.5 × 10^5^ LN229 cells/well were seeded in 6-well plates, and after 24 h, cells were transfected with control siRNA or siRNAs against *CLTC*. Next, 48 h after transfection, 4 × 10^4^ cells/well were re-plated on 12-mm coverslips in 24-well plates, and 24 h later transfected again with the same siRNA. Cells were analyzed 72 h after second transfection and silencing efficiency was controlled by WB.

### Western blot (WB) and quantitative real-time PCR (qRT-PCR)

WB and qRT-PCR analyses were performed as described elsewhere [111]. Primers used for qRT-PCR analysis of gene expression are listed in Table S3. Data were quantified using the Data Assist v2.0 software (Applied Biosystems) and normalized to the level of ACTB (actin) mRNA.

### TIRF live-cell microscopy

Live-cell imaging of LN229 cells expressing AXL-EGFP together with mRFP-CLC or mCherry-PICALM was performed using a Deltavision OMX V4 (GE Healthcare) using a 60 × TIRF objective. Images were acquired every 20 s up to 10 min after the ligand administration and were further deconvolved as described elsewhere [112]. The first image was acquired approximately 1 min ± 30 s after GAS6 addition due to required correction of z position.

### Generation of CRISPR-Cas9-mediated KO LN229 cells and LN229 stably expressing BirA*-HA or AXL-BirA*-HA

Cell lines were established via lentiviral transduction of LN229 cells, as described elsewhere [31, 111]. To knockout *DNM2*, *EPS15*, *EPS15L1*, *NUMB*, *CAV1, FLOT1*, *SNX1,* and *SNX2* gRNA sequences from the Brunello library [113] were used (Table S1). Cells expressing non-targeting gRNAs, as well as *AXL* and *TYRO3* KO cell lines were established previously [31]. LN229 cells stably expressing AXL-BirA*-HA or BirA*-HA were generated previously [31].

### Proximity-dependent biotin identification (BioID)

BioID was performed as previously described [31]. Obtained samples were analyzed by WB.

## Supplementary Information

Below is the link to the electronic supplementary material.

Supplementary file1 (MP4 1806 KB)

Supplementary file2 (MP4 3300 KB)

Supplementary file3 (PDF 4559 KB)

## Data Availability

All data generated or analyzed during this study are included in this published article and its supplementary materials. Raw data of all quantitatively analyzed experiments are available from the corresponding author on reasonable request.
