# Supplementary material for: Endocytic trafficking of GAS6–AXL complexes is associated with sustained AKT activation
Source: Cell Mol Life Sci. 2022 May 27;79(6):316. doi: 10.1007/s00018-022-04312-3 (PMC9135597; doi:10.1007/s00018-022-04312-3)
Supplement: Supplementary file 3 — Supplementary file3 (PDF 4559 KB) [file 18_2022_4312_MOESM3_ESM.pdf]

Supplementary Figures and their legends

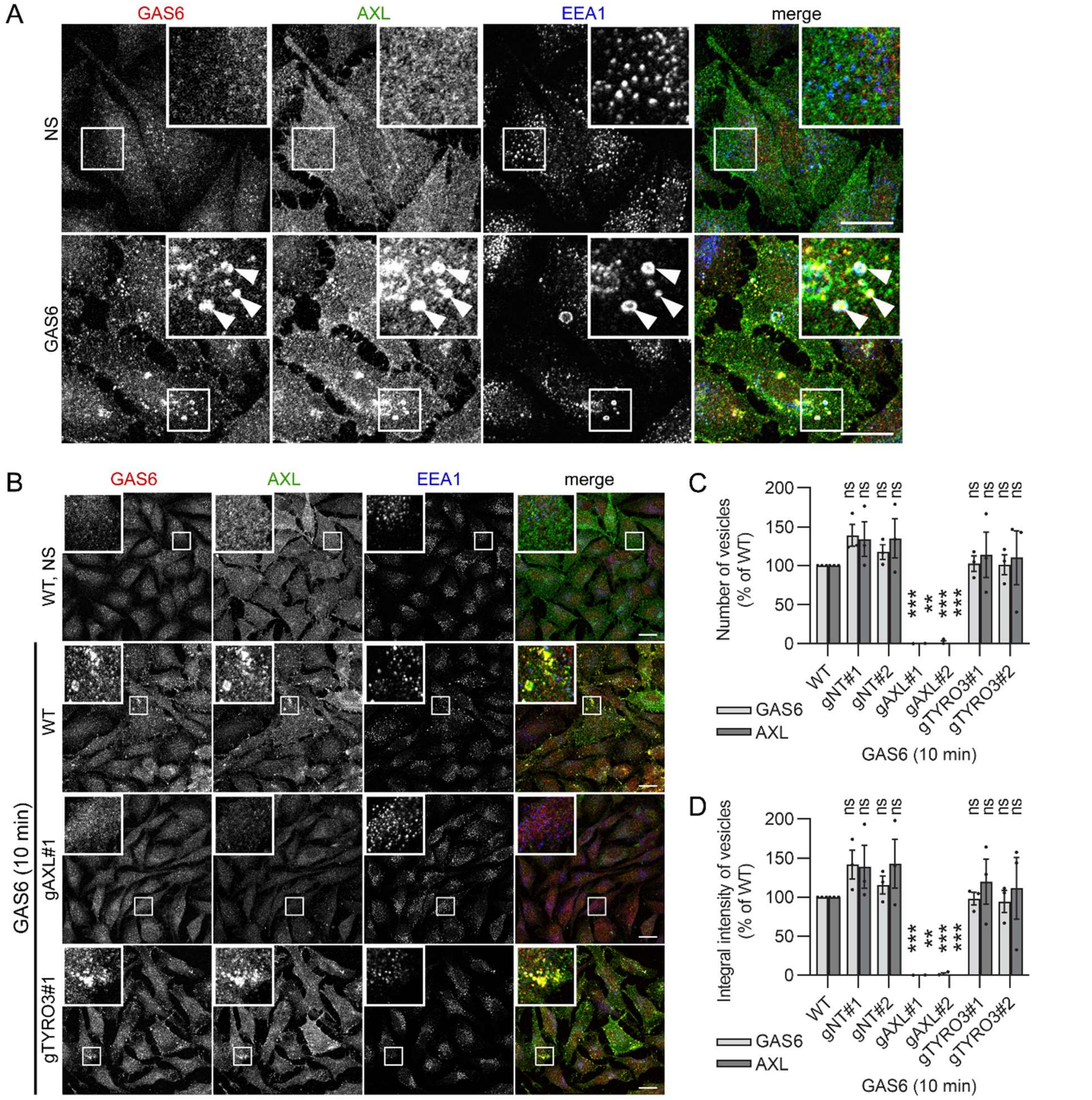

Fig. S1 Knockout of AXL, but not TYRO3, inhibits GAS6 internalization. A Confocal images showing GAS6–AXL internalization in LN229 cells. Serum-starved cells were stimulated with GAS6 for 5 min. Arrowheads indicate macropinosomes. **B** Confocal images showing GAS6–AXL internalization upon knockout of *AXL* or *TYRO3* in LN229 cells. Two gRNAs targeting AXL (gAXL#1 and gAXL#2) and targeting TYRO3 (gTYRO3#1 and TYRO3#2) were used. CRISPR-Cas9-edited LN229 cells with two non-targeting gRNAs (gNT#1 and gNT#2) served as controls. Serum-starved cells were stimulated with GAS6 for 10 min. **C, D** Quantification of number (C) and integral fluorescence intensity (D) of GAS6- and AXL-positive

vesicles in knockouts of *AXL* and *TYRO3* (representative confocal images shown in B), n=3. Student's one-sample *t* test, **p≤0.01, ***p≤0.001, ns- non-significant (p>0.05).

Data information: Insets in confocal images show magnified views of boxed regions in the main images. Scale bars: 20 µm. For data quantification approximately 150 cells were analyzed per experiment. Each dot represents data from one independent experiment, whereas bars represent the means ± SEM from n experiments. WT- wild type LN229 cells, NS- non-stimulated cells, GAS6- GAS6-stimulated cells.

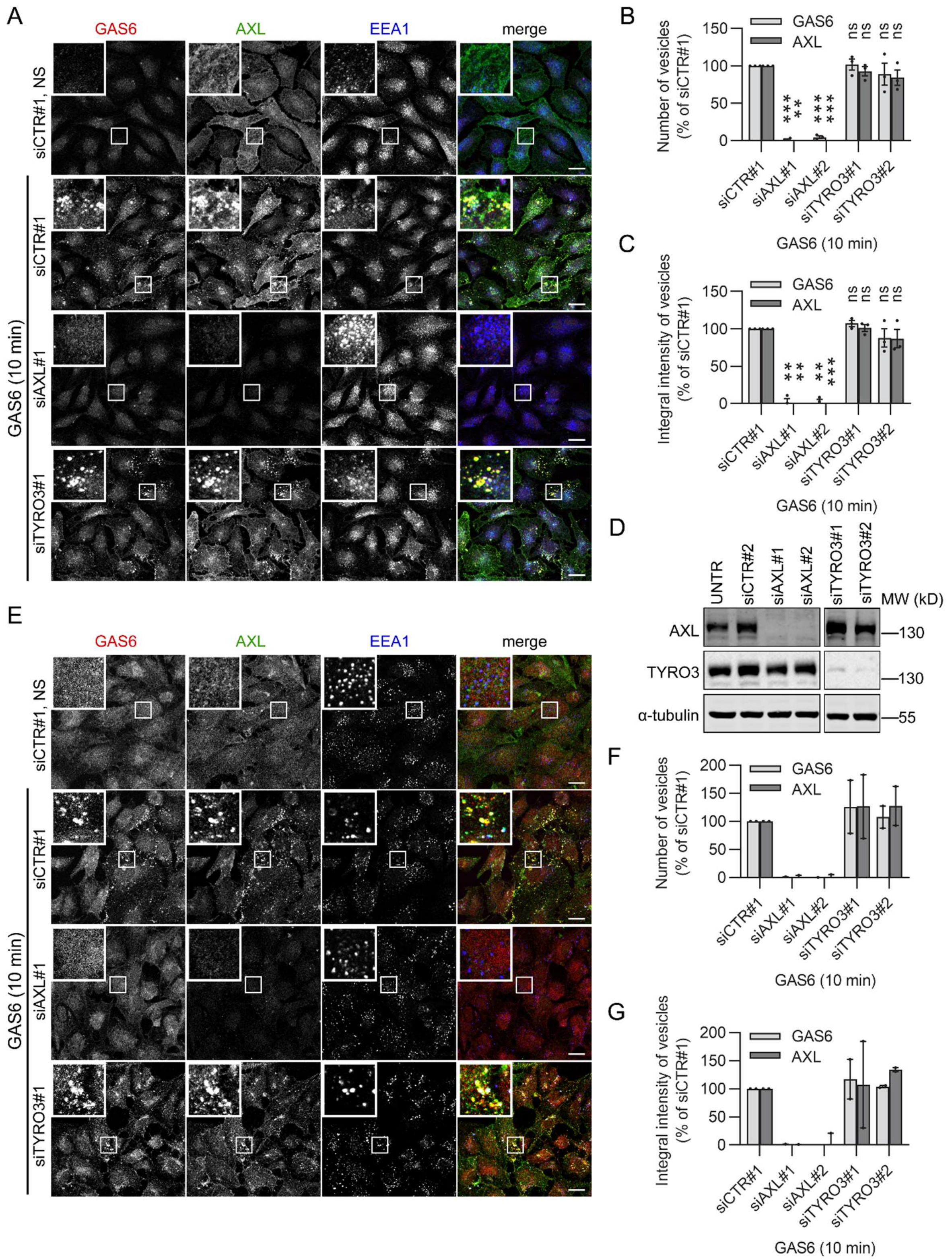

Fig. S2 Depletion of AXL, but not TYRO3, inhibits GAS6 internalization. A Confocal images showing GAS6–AXL internalization upon silencing of *AXL* or *TYRO3* in LN229 cells.

Two siRNAs targeting *AXL* (siAXL#1 and siAXL#2) and targeting *TYRO3* (siTYRO3#1 and siTYRO3#2) were used. LN229 cells transfected with non-targeting siRNA (siCTR#1) served as controls. 72 h after transfection serum-starved cells were stimulated with GAS6 for 10 min **B, C** Quantification of number (B) and integral fluorescence intensity (C) of GAS6- and AXL-positive vesicles in LN229 cells depleted of AXL or TYRO3 cells, n=3 (representative confocal images shown in A). Student's one-sample *t* test, **p≤0.01, ***p≤0.001, ns - non-significant (p>0.05). **D** Western blot showing efficiency of *AXL* or *TYRO3* silencing in LN229 cells. Cells were transfected as described in A, UNTR- non-transfected cells. α-Tubulin served as a loading control. **E** Confocal images showing GAS6–AXL internalization upon knockdown of *AXL* and *TYRO3* in SKOV3 cells. Cells were transfected and stimulated with GAS6 as described in A. **F, G** Quantification of number (F) and integral fluorescence intensity (G) of GAS6- and AXL-positive vesicles in SKOV3 cells depleted of AXL or TYRO3, n=2 (representative confocal images shown in E).

Data information: Insets in confocal images show magnified views of boxed regions in the main images. Scale bars: 20 μm. For data quantification approximately 150 cells were analyzed per experiment. Each dot represents data from one independent experiment, whereas bars represent the means ± SEM from n experiments. NS- non-stimulated cells, GAS6- GAS6-stimulated cells.

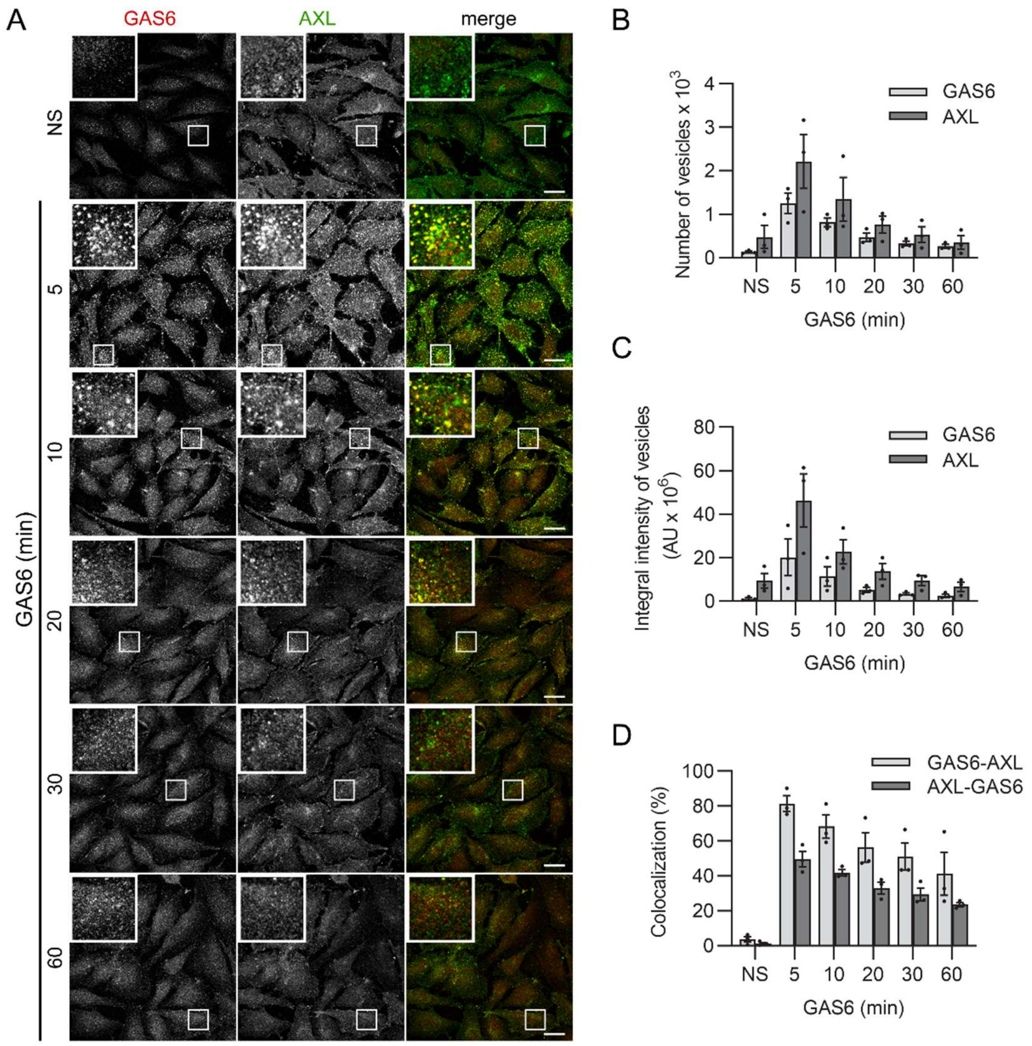

Fig. S3 Pulse-chase stimulation with GAS6 triggers rapid internalization of GAS6–AXL complexes in LN229 cells. A Confocal images showing the kinetics of internalization of GAS6 and AXL in LN229 cells after pulse-chase stimulation with GAS6. Serum-starved cells were incubated on ice with GAS6, and after removing of unbound ligand, endocytosis was allowed to proceed at 37 °C for the indicated time periods. **B, C, D** Quantification of number (B), integral fluorescence intensity (C) and colocalization (D) between GAS6- and AXL-positive vesicles (representative confocal images shown in A), n=3. GAS6-AXL- percentage of GAS6-positive vesicles overlapping with AXL-positive vesicles, AXL-GAS6- percentage of AXL-positive vesicles overlapping with GAS6-positive vesicles.

Data information: Insets show magnified views of boxed regions in the main images. Scale bars: 20 μm. For data quantification approximately 150 cells were analyzed per experiment. Each dot represents data from one independent experiment, whereas bars represent the means ± SEM from n experiments, AU- arbitrary units.

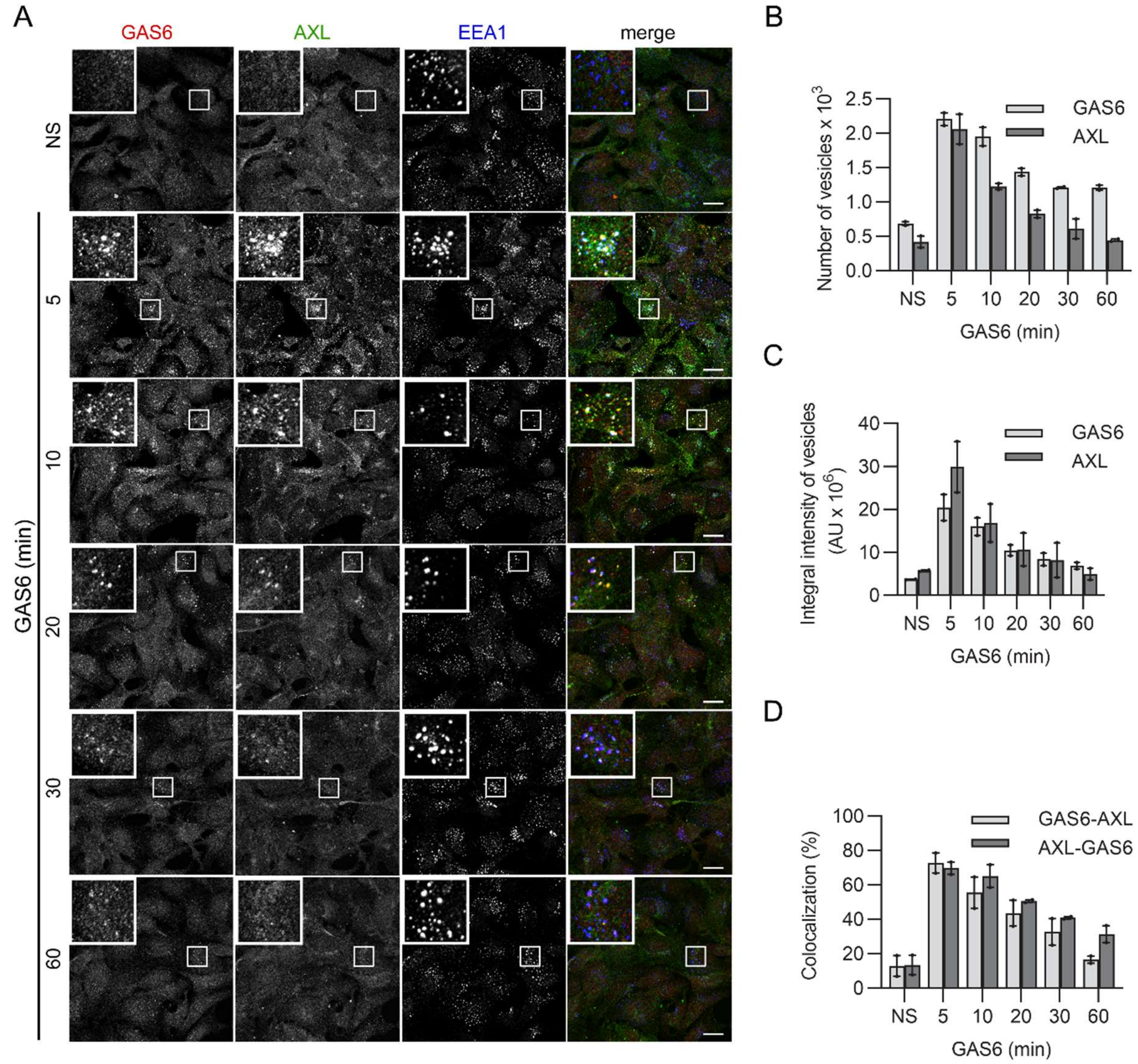

Fig. S4 Pulse-chase stimulation with GAS6 triggers rapid internalization of GAS6–AXL complexes in SKOV3 cells. A Confocal images showing the kinetics of internalization of GAS6 and AXL in SKOV3 cells after pulse-chase stimulation with GAS6. Serum-starved cells were stimulated with GAS6 as described in Fig. S3A. **B, C, D** Quantification of number (B), integral fluorescence intensity (C) and colocalization (D) between of GAS6- and AXL-positive vesicles (representative confocal images shown in A), n=2. GAS6-AXL- percentage of GAS6-positive vesicles overlapping with AXL-positive vesicles, AXL-GAS6- percentage of AXL-positive vesicles overlapping with GAS6-positive vesicles.

Data information: Insets show magnified views of boxed regions in the main images. Scale bars: 20 µm. For data quantification approximately 150 cells were analyzed per experiment. Each dot represents data from one independent experiment, whereas bars represent the means ± SEM from n experiments, AU- arbitrary units.

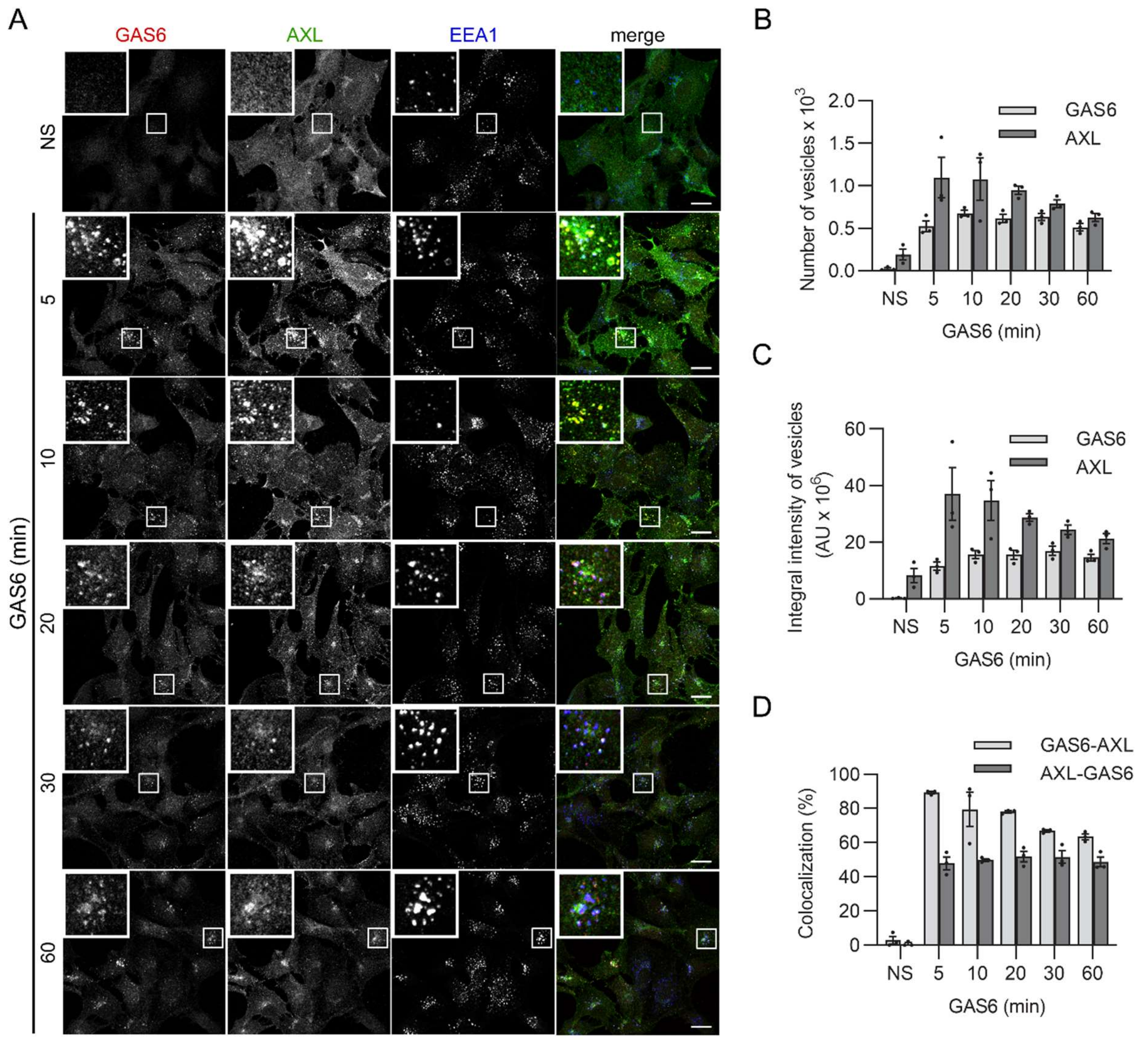

Fig. S5 Continuous stimulation of SKOV3 cells with GAS6 triggers rapid internalization of GAS6–AXL complexes. A Confocal images showing the kinetics of internalization of GAS6 and AXL in SKOV3 cells after continuous stimulation with GAS6. Cells were serum-starved and stimulated with GAS6 for the indicated time periods. **B, C, D** Quantification of number (B), integral fluorescence intensity (C) and colocalization (D) between GAS6- and AXL-positive vesicles (representative confocal images shown in A), n=3. GAS6-AXL- percentage of GAS6-positive vesicles overlapping with AXL-positive vesicles, AXL-GAS6- percentage of AXL-positive vesicles overlapping with GAS6-positive vesicles.

Data information: Insets show magnified views of boxed regions in the main images. Scale bars: 20 µm. For data quantification approximately 150 cells were analyzed per experiment. Each dot represents data from one independent experiment, whereas bars represent the means ± SEM from n experiments, AU- arbitrary units.

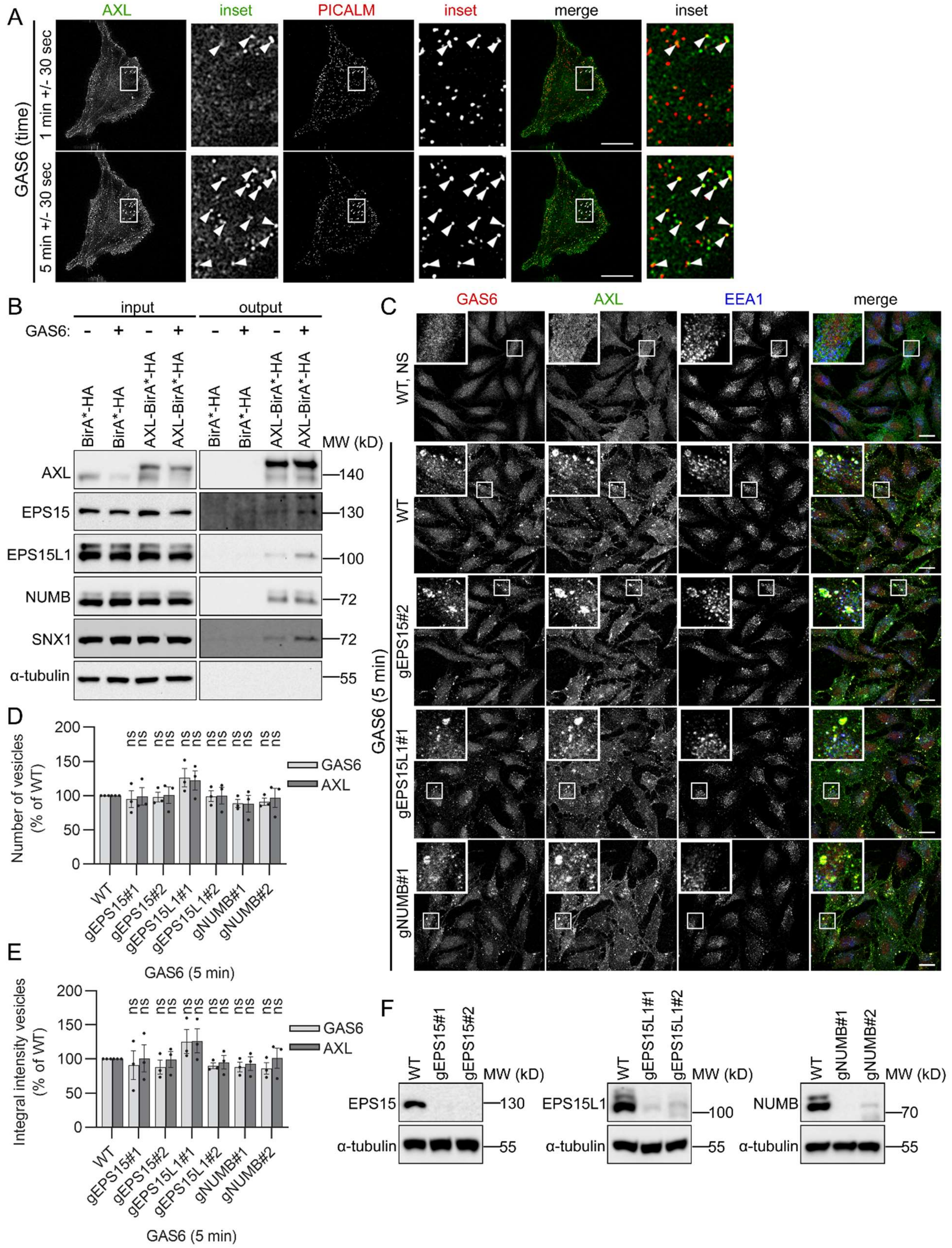

Fig. S6 AXL colocalizes with PICALM but depletion of EPS15, EPS15L1 or NUMB does not affect GAS6–AXL endocytosis. A Total internal reflection fluorescence (TIRF) analysis of

living LN229 cells expressing AXL-EGFP and mCherry-PICALM. Serum-starved cells were imaged every 20 s up to 10 min after GAS6 addition. Representative frames from Movie S2 are shown. Arrowheads indicate structures positive for both AXL and PICALM. **B** Western blot showing proteins identified by proximity dependent biotin identification as AXL proximity interactors. LN229 expressing AXL-BirA*-HA and BirA*-HA were incubated with biotin in the presence or absence of GAS6. Samples from cell lysates containing biotinylated proteins were analyzed by WB before (input) and after (output) pulldown with streptavidin-coated magnetic beads. α-Tubulin was used as a loading control. **C** Confocal images showing GAS6–AXL internalization upon knockout of *EPS15*, *EPS15L1* or *NUMB* in LN229 cells. Two gRNA targeting *EPS15* (gEPS15#1 and gEPS15#2), *EPS15L1* (gEPS15L1#1 and gEPS15L1#2) or *NUMB* (gNUMB#1 and gNUMB#2) were used. WT LN229 cells served as controls. Serum-starved cells were stimulated with GAS6 for 5 min. **D, E** Quantification of number (D) and integral fluorescence intensity (E) of GAS6- and AXL-positive vesicles in knockouts of *EPS15, EPS15L1* or *NUMB* (representative confocal images shown in C), n=3. Student's one-sample *t* test, ns - non-significant ($p>0.05$). **F** Western blot showing the efficiency of CRISPR-Cas9-mediated KO of *EPS15, EPS15L1* and *NUMB*. α-Tubulin was used as a loading control.

Data information: Insets in confocal images show magnified views of boxed regions in the main images. Scale bars: 20 µm. For data quantification approximately 150 cells were analyzed per experiment. Each dot represents data from one independent experiment, whereas bars represent the means ± SEM from n experiments. WT- wild type LN229 cells, NS- non-stimulated cells, GAS6- GAS6-stimulated cells.

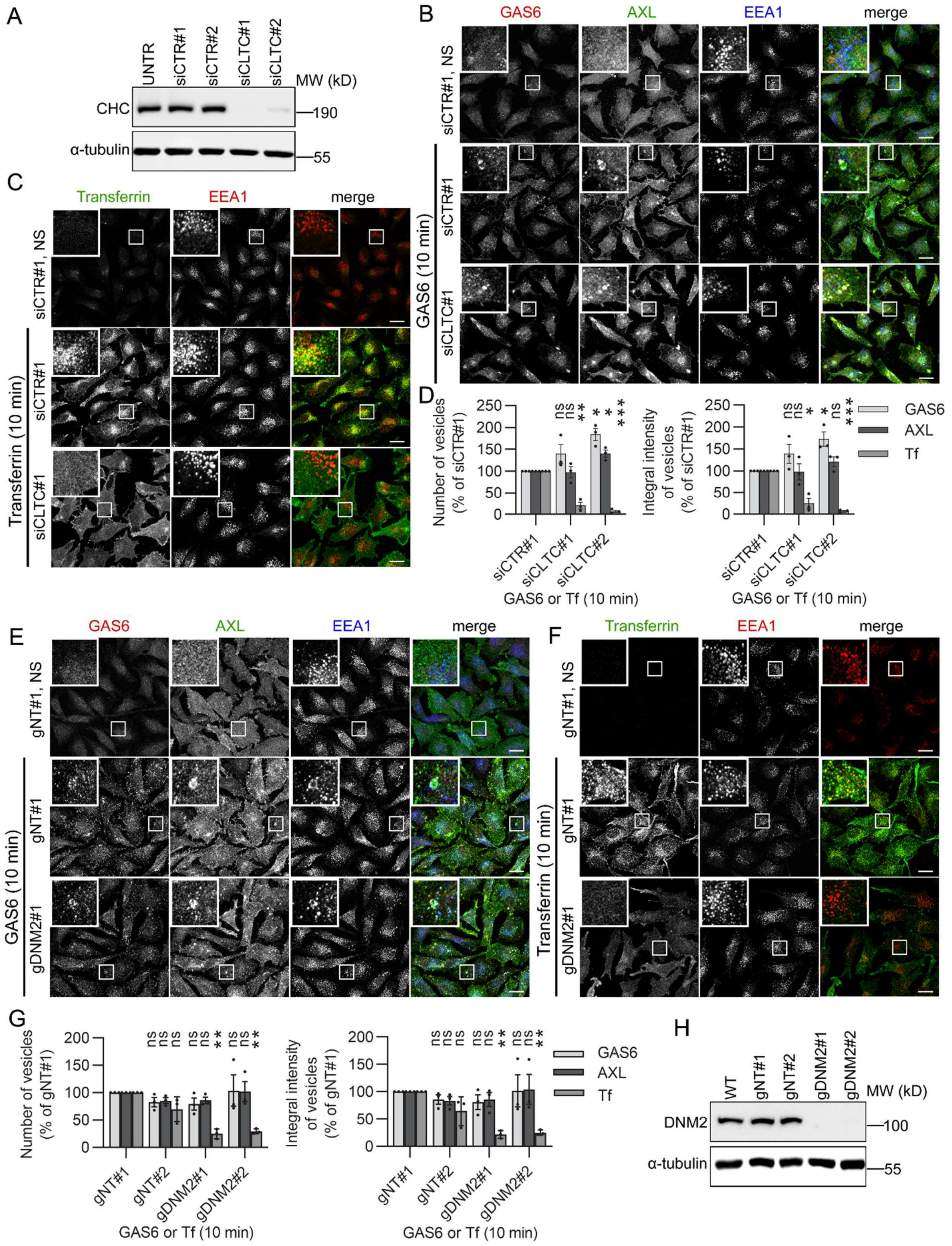

Fig. S7 Depletion of CHC or DNM2 does not decrease GAS6–AXL endocytosis, in contrast to transferrin, a well-established cargo for CME. A Western blot showing efficiency of *CLTC*

silencing. Cells were transfected twice with two non-targeting siRNAs (siCTR#1 and siCTR#2) and two siRNAs targeting *CLTC* (siCLTC#1 and siCLTC#2) with 72 h break between transfections. 72 h after second transfection cells were lysed. UNTR- non-transfected cells. α-Tubulin served as a loading control. **B, C** Confocal images showing GAS6–AXL (B) or transferrin (Tf, C) internalization upon knockdown of *CLTC* in LN229 cells. Two siRNAs targeting *CLTC* (siCLTC#1 and siCLTC#2) were used. LN229 cells transfected with non-targeting siRNA (siCTR#1) served as control. Cells were transfected as described in A. 72 h after second transfection serum-starved cells were stimulated with GAS6 (B) or Tf (C) for 10 min. **D** Quantification of number and integral fluorescence intensity of GAS6-, AXL- and Tf-positive vesicles in LN229 cells upon knockdown of *CLTC* (representative confocal images shown in B and C), n=3. Student's one-sample *t* test, $^*p \leq 0.05$, $^{**}p \leq 0.01$, $^{***}p \leq 0.001$, ns - non-significant ($p>0.05$). **E, F** Confocal images showing GAS6–AXL (E) or Tf (F) internalization upon knockout of *DNM2* in LN229 cells. Two gRNAs targeting DNM2 (gDNM2#1 and gDNM2#2) were used. CRISPR-Cas9-edited LN229 cells with two non-targeting gRNAs (gNT#1 and gNT#2) served as controls. Serum-starved cells were stimulated with GAS6 (E) or Tf (F) for 10 min. **G** Quantification of number and integral fluorescence intensity of GAS6-, AXL- and Tf-positive vesicles in knockouts of *DNM2* (representative confocal images shown in E and F), n=3. Student's one-sample *t* test, $^{**}p \leq 0.01$, ns - non-significant ($p>0.05$). **H** Western blot showing the efficiency of *DNM2* knockout. α-Tubulin served as a loading control.

Data information: Insets in confocal images show magnified views of boxed regions in the main images. Scale bars: 20 µm. For data quantification approximately 150 cells were analyzed per experiment. Each dot represents data from one independent experiment, whereas bars represent the means ± SEM from n experiments. WT- wild type LN229 cells, NS- non-stimulated cells, GAS6- GAS6-stimulated cells, Transferrin/Tf- transferrin-stimulated cells, GAS6 or Tf- GAS6- or transferrin-stimulated cells.

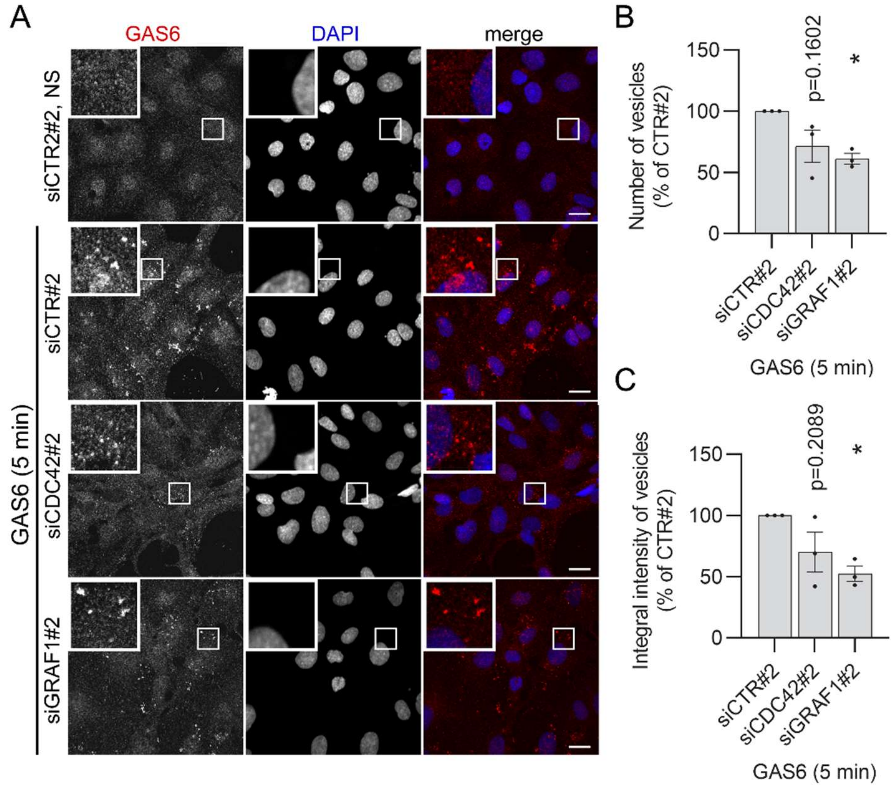

Fig. S8 Depletion of CDC42 or GRAF1 reduces GAS6 internalization in SKOV3 cells. A Confocal images showing GAS6 internalization upon depletion of CDC42 or GRAF1 in SKOV3 cells. siRNA targeting *CDC42* (siCDC42#2) or *GRAF1* (siGRAF1#2) were used for depletion. SKOV3 cells transfected with non-targeting siRNA (siCTR#2) served as control. 72 h after transfection serum-starved cells were stimulated with GAS6 for 5 min. **B, C** Quantification of number (B) and integral fluorescence intensity (C) of GAS6-positive vesicles in SKOV3 cells depleted of CDC42 or GRAF1 (representative confocal images shown in A), n=3. Student's one-sample *t* test, *p≤0.05.

Data information: Insets in confocal images show magnified views of boxed regions in the main images. Scale bars: 20 µm. For data quantification approximately 150 cells were analyzed per experiment. Each dot represents data from one independent experiment, whereas bars represent the means ± SEM from n experiments. NS- non-stimulated cells, GAS6- GAS6-stimulated cells.

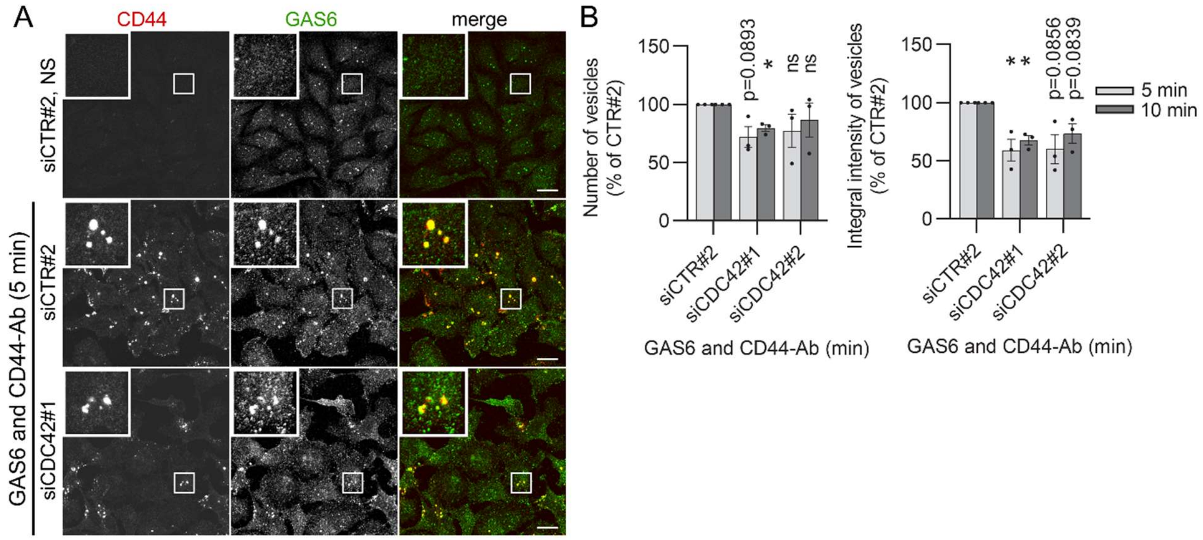

Fig. S9 Depletion of CDC42 decreases GAS6-induced uptake of CD44. A Confocal images showing CD44 internalization upon knockdown of *CDC42* in LN229 cells. Two siRNAs targeting *CDC42* (siCDC42#1 and siCDC42#2) were used. LN229 cells transfected with non-targeting siRNAs (siCTR#2) served as control. 72 h after transfection serum-starved cells were stimulated with GAS6 and agonistic antibody recognizing CD44 (CD44-Ab) for 5 min. **B** Quantification of number and integral fluorescence intensity of CD44-positive vesicles in cells depleted of CDC42 and stimulated with GAS6 and CD44-Ab for 5 or 10 min (representative confocal images shown in A), n=3. Student's one-sample *t* test, *p≤0.05, ns - non-significant (p>0.05).

Data information: Insets in confocal images show magnified views of boxed regions in the main images. Scale bars: 20 µm. For data quantification approximately 150 cells were analyzed per experiment. Each dot represents data from one independent experiment, whereas bars represent the means ± SEM from n experiments. NS- non-stimulated cells, GAS6 and CD44-Ab- cells stimulated with GAS6 and CD44-Ab.

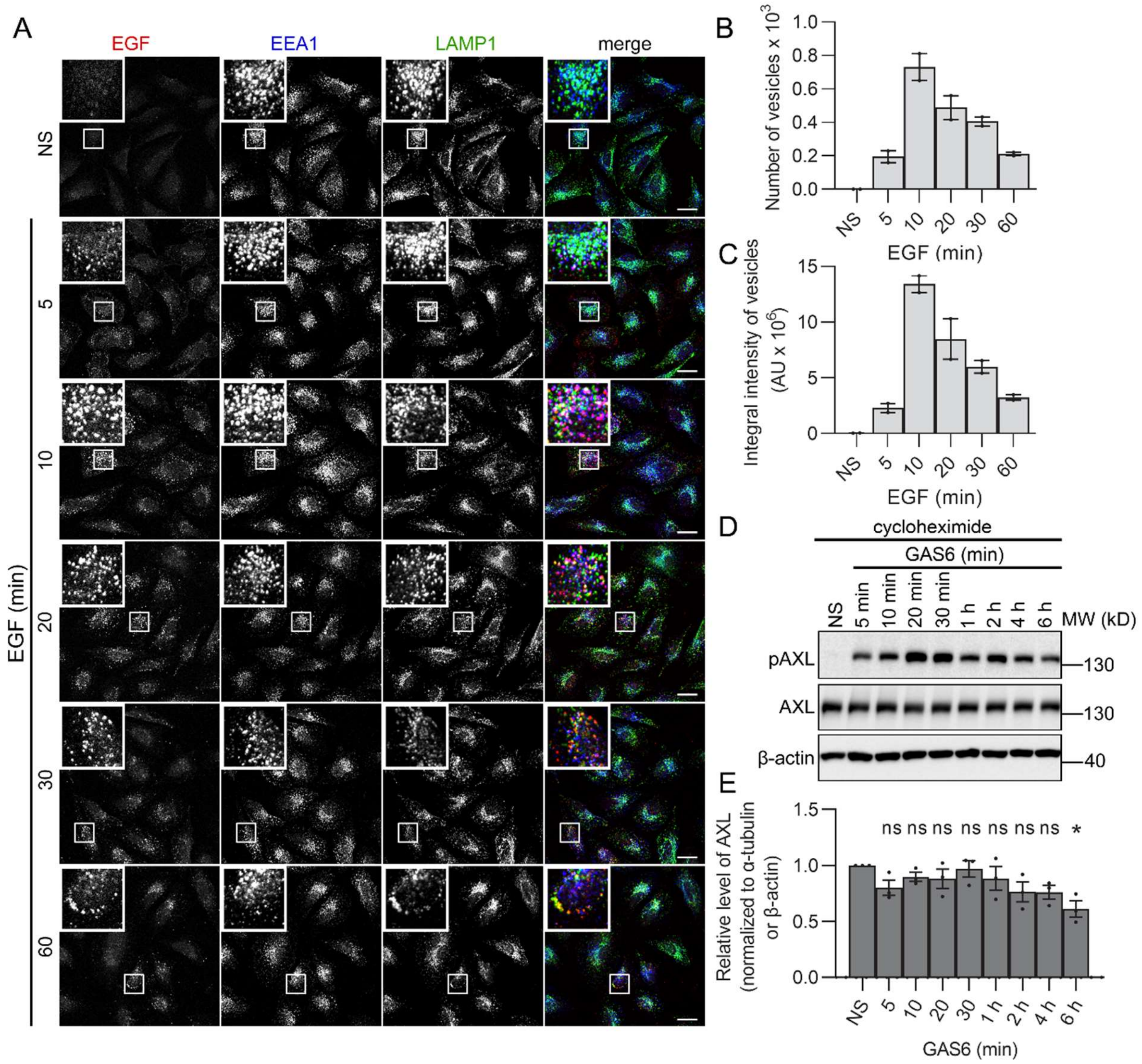

Fig. S10 EGFR displays high colocalization with EEA1 and LAMP1, and lower colocalization with GAS6 and AXL. Pulse-chase stimulation with GAS6 does not trigger AXL degradation. A Confocal images showing the kinetics of internalization of EGF and its colocalization with EEA1 and LAMP1 in LN229 cells. Serum-starved cells were stimulated with EGF for the indicated time periods. **B, C** Quantification of number (B) and integral fluorescence intensity (C) of EGF-positive vesicles (representative confocal images shown in A), n=2. **D** Western blot showing GAS6-induced phosphorylation of AXL (P-AXL, Y702) and total level of AXL after pulse-chase stimulation with GAS6. Serum-starved LN229 cells were pre-treated with cycloheximide and next stimulated with GAS6 as described in Fig. S3A. β-Actin served as a loading control. **E** Graph showing the densitometric analysis of AXL levels shown in D, normalized to α-tubulin, n=3. Student's one-sample t-test, *$p \leq 0.05$, ns - non-significant ($p > 0.05$).

Data information: Insets in confocal images show magnified views of boxed regions in the main images. Scale bars: 20 μm. For data quantification approximately 150 cells were analyzed per

experiment. Each dot represents data from one independent experiment, whereas bars represent the means ± SEM from n experiments. NS- non-stimulated cells, EGF- EGF-stimulated cells, GAS6- GAS6-stimulated cells, AU- arbitrary units.

Supplementary Movie legends

Movie S1 and S2. AXL colocalizes with CLC (Movie S1) and PICALM (Movie S2). Serum starved LN229 cells expressing AXL-EGFP and mRFP-CLC (Movie S1) or mCherry-PICALM (Movie S2) were monitored by total internal reflection fluorescence (TIRF) microscopy for 10 min after addition of GAS6. The first image was acquired approximately 1 min +/- 30 s after GAS6 addition due to required correction of z position. The rest of images were acquired every 20 s. Scale bars: 20 μm.

Supplementary Tables and their legends:

Table S1. gRNAs used for CRISPR-Cas9-mediated gene inactivation. Two 20-bp-long single-guide RNAs (gRNAs) were selected from the Brunello library [104] and appropriate pairs of DNA oligonucleotides were designed as described elsewhere [98].

gRNA name	Oligonucleotide name	Sequence (5'-3')
gDNM2#1	gDNM2#1-F	CACCGCGTGTGGCGAGTAGACTCGA
	gDNM2#1-R	AAACTCGAGTCTACTCGCCACACG
gDNM2#2	gDNM2#2-F	CACCGTCAATCGCATCTTCCACGAG
	gDNM2#2-R	AAACCTCGTGGAAGATGCGATTGAC
gCAV1#1	gCAV1#1-F	CACCGGCACACCAAGGAGATCGACC
	gCAV1#1-R	AAACGGTCGATCTCCTTGGTGTGCC
gFLOT1#1	gFLOT1#1-F	CACCGAGACGTTAGAGGGCCACCAG
	gFLOT1#1-R	AAACCTGGTGGCCCTCTAACGTCTC
gFLOT1#2	gFLOT1#2-F	CACCGAAAGGTTTACACTCGCCATG
	gFLOT1#2-R	AAACCATGGCGAGTGTAAACCTTTC
gEPS15#1	gEPS15#1-F	CACCGGAGGTTCCACTGATTAGCAA
	gEPS15#1-R	AAACTTGCTAATCAGTGGAACCTCC
gEPS15#2	gEPS15#2-F	CACCGTTATGCGACACAAAGGACTG
	gEPS15#2-R	AAACCAGTCCTTTGTGTCGCATAAC
gEPS15L1#1	gEPS15L1#1-F	CACCGGGATCGAGATGAGTTCGCTG
	gEPS15L1#1-R	AAACCAGCGAACTCATCTCGATCCC
gEPS15L1#2	gEPS15L1#2-F	CACCGTCCGTACCTGTGTTTGCTTG
	gEPS15L1#2-R	AAACCAAGCAAACACAGGTACGGAC
gNUMB#1	gNUMB#1-F	CACCGATCCTCATGCCATCCCACGC
	gNUMB#1-R	AAACGCGTGGGATGGCATGAGGATC
gNUMB#2	gNUMB#2-F	CACCGCTATCGTCTGGTCAACTATG
	gNUMB#2-R	AAACCATAGTTGACCAGACGATAGC
gSNX1	gSNX1-F	CACCGAGCCTACAAAGTTACAACAC
	gSNX1-R	AAACGTGTTGTAACTTTGTAGGCTC
gSNX2	gSNX2-F	CACCGCAGCACTGTCTCCACCCTAG
	gSNX2-R	AAACCTAGGGTGGAGACAGTGCTGC

Table S2. Small interfering RNA (siRNA) oligonucleotides. Ambion Silencer Select siRNAs were from Thermo Fisher Scientific. As negative control non-specific Silencer Select siRNA oligonucleotides (siCTR#1 4390846 and siCTR#2 AM4615) were used.

Name	Catalog no.	Sequence (5'-3')
siAXL#1	s1845	GGAACUGCAUGCUGAAUGAtt
siAXL#2	s1846	GGGUGGAGGUUAUCCUGAAtt
siAXL#3	s1847	AGCGAGAUUUAUGACUAUtt
siTYRO3#1	s14544	GAGCUUUACUUGUCUGCGAtt
siTYRO3#2	s14545	CAGUGACUGUCGGUACAUAtt
siTYRO3#3	s14546	CAAGCGACAUUGAAGAGUUtt
siCDC42#1	s2765	UGGUGCUGUUGGUAAAACAtt
siCDC42#2	s2767	CAGUUAUGAUUGGUGGAGAtt
siCLTC#1	s475	GGUUGCUCUUGUUACGGAUtt
siCLTC#2	s476	CGGUUGCUCUUGUUACGGAtt
siGRAF1#1	s23013	GAGCAAGGGCUGUAUCGAAtt
siGRAF1#2	s23015	GGAUACGGAUGAUUGAGAAtt

Table S3. Primers used in qRT-PCR analysis of gene expression.

Gene name	Oligonucleotide name	Sequence (5'-3')
ACTB	ACTB-F	CAGGTCATCACCATTGGCAAT
	ACTB-R	TCTTTGCGGATGTCCACGT
GRAF1	GRAF1-F	TAAGAATGCTTCCAGGACCACTC
	GRAF1-R	GCTGTAACATCTGCCGATTTTTC
